# Effectiveness of an artificial intelligence-based self-management intervention to support sick-listed individuals with musculoskeletal disorders in Norway: rationale and protocol for the SmaRTWork randomised controlled trial

**DOI:** 10.1136/bmjopen-2026-122680

**Published:** 2026-08-18

**Authors:** Lene Aasdahl, Kerstin Bach, Tom Ivar Lund Nilsen, Stuart Gallina Ottersen, Nina E Klevanger, Christoph Eder, Cecilie Krage Øverås, Fredrik Granviken, Julia Debik, Lars Christian Naterstad Lervik, Heidi Fossen, Anita Formo Bones, Sigmund Gismervik, Karen W Hara, Paul Jarle Mork

**Affiliations:** 1 Department of Public Health and Nursing, Norwegian University of Science and Technology, Trondheim, Trøndelag, Norway; 2 Unicare Helsefort Rehabilitation Centre, Rissa, Norway; 3 Department of Computer Science, Norwegian University of Science and Technology, Trondheim, Norway; 4 Et Liv i Bevegelse (ELiB), The Norwegian Chiropractors’ Research Foundation, Oslo, Norway; 5 Clinic of Rehabilitation, St Olavs Hospital Universitetssykehuset i Trondheim, Trondheim, Norway; 6 Hallset Legesenter AS, Trondheim, Norway; 7 General Practice Research Unit, Norwegian University of Science and Technology, Trondheim, Norway; 8 The Norwegian Labour and Welfare Service of Trøndelag, Trondheim, Norway; 9 Norwegian Cancer Society, Trondheim, Norway

**Keywords:** Pain management, Health Workforce, Musculoskeletal disorders

## Abstract

**Introduction:**

Long-term sickness absence represents a major public health challenge with far-reaching consequences for both individuals and society. Musculoskeletal disorders (MSDs) are among the leading causes of sickness absence. Although mobile apps show promise for supporting self-management of MSDs, high-quality evidence remains limited, and outcomes related to work participation are rarely examined. This trial aims to evaluate whether an individually tailored, app-based self-management intervention (SmaRTWork), provided in addition to usual care, improves return-to-work compared with usual care alone among individuals on sick leave due to MSDs.

**Methods and analysis:**

We will conduct a randomised controlled trial with two parallel arms: 1) SmaRTWork in addition to usual care and 2) usual care alone. Individuals 20–59 years old who have been sick-listed for <12 weeks due to back, neck or widespread pain will be recruited through clinicians in primary health care. Eligible participants are randomised 1:1 to one of the two arms. We aim to include 298 participants, 149 in each arm. The primary outcome is time to sustainable return-to-work, defined as one consecutive month without receiving medical benefits, assessed over a 12-month period using registry data. Secondary outcomes include health-related measures assessed at baseline, 3, 6 and 12 months. The trial will also incorporate qualitative studies, a process evaluation and a health economic evaluation.

**Ethics and dissemination:**

The trial is approved by the Committees for Clinical Trials of Pharmaceuticals and Medical Devices (Ref. 563919). Results will be published in peer-reviewed journals and presented at national and international conferences.

**Trial registration:**

NCT07034040.

**STRENGTHS AND LIMITATIONS OF THIS STUDY**

- This study uses a randomised controlled trial design to evaluate the effectiveness of SmaRTWork, an individually tailored, app-based self-management intervention aimed at supporting return-to-work among individuals sick-listed due to musculoskeletal disorders.
- Qualitative studies and a process evaluation will be conducted to gain insight into participants’ experiences with the SmaRTWork app and to identify key facilitators and barriers to its implementation.
- A health economic evaluation will be conducted to assess cost-effectiveness, cost-utility and cost-benefit from both healthcare and societal perspectives.
- A potential selection bias related to technological competence may limit the external validity of the study.
- Participants are not blinded, only researchers performing the analyses (single-blinded study).

## Introduction

 Long-term sickness absence represents a major public health challenge with far-reaching consequences for both individuals and society.^1 2^ Its negative impact is substantial, including high economic costs, individual suffering and loss of workforce.^2^ Moreover, the literature suggests that sick leave tends to become increasingly complex over time and the likelihood of returning to work decreases as absence is prolonged,^1 3^ indicating that early intervention is likely to be important.

Musculoskeletal disorders (MSDs) are the leading cause of sickness absence in the European Union.^2^ Central in the recommendations for treatment of MSDs is self-management.^4 5^ This approach does not replace the role of clinicians but aims to empower patients, in contrast to earlier models of passive provider-led care.^6 7^ Although definitions vary, self-management generally refers to the ability to cope and manage daily life despite having a chronic condition.^7^ A key element in self-management programmes is to enhance self-efficacy, defined as the “belief in one’s abilities to organize and execute the courses of action required to produce given attainments”.^8^ Accordingly, interventions for MSDs often include education, advice to stay active, exercise and some form of cognitive behavioural therapy.^9^ However, health professionals often lack the time and training to deliver self-management support^10 11^, and adherence to self-management over time can be challenging for many patients without support.^12^ To support self-management and increase adherence, smartphone app-based interventions have emerged as a promising approach during the last decade.^13^ Although some evidence suggests that such interventions may improve self-management of MSDs, the evidence base is limited by few high-quality studies and considerable heterogeneity in intervention content.^14–16^ Moreover, work participation outcomes are rarely examined, and there have been explicit calls for interventions specifically developed for individuals sick-listed due to MSDs.^13–15 17 18^

In a previous randomised controlled trial (RCT), we found that an artificial intelligence-based app (selfBACK) for supporting self-management of low back pain in a primary care setting produced a small, clinically uncertain reduction in pain-related disability.^19^ Another RCT of the selfBACK app was conducted in a specialist care setting without showing an effect.^20^ However, a recent systematic review and meta-analysis found a larger effect on disability than on pain intensity when evaluating digital self-management interventions for low back pain.^14^ This aligns with the aim of self-management, which is to enhance coping rather than to eliminate symptoms.^11^ This is also consistent with the work disability paradigm, which views low back pain as not only a clinical condition but also a psychosocial and work-related issue and that pain relief is not a prerequisite for returning to work.^21^ However, the evidence on the effectiveness of app-based self-management interventions for improving return-to-work among individuals sick-listed due to MSDs remains limited.^14 17^ To prepare this RCT, intervention mapping was used to further develop and refine the selfBACK app to support self-management and facilitate return-to-work for individuals sick-listed due to MSDs.

The primary aim of this RCT is to evaluate whether an individually tailored app-based self-management intervention (SmaRTWork), provided in addition to usual care, improves return-to-work compared with usual care alone among individuals on sick leave due to MSDs. A secondary aim is to evaluate the effectiveness of the SmaRTWork intervention on health-related measures such as pain and sleep. The RCT will also incorporate qualitative studies, a process evaluation and a health economic evaluation.

## Methods

### Trial design

We will conduct an RCT with two parallel arms to evaluate the effectiveness of the SmaRTWork intervention. Additionally, the project includes qualitative studies, a process evaluation and a health economic evaluation. Results will be reported according to the Consolidated Standards of Reporting Trials (CONSORT) statement^22^ and the CONSORT-AI extension,^23^ and this protocol is reported according to the Standard Protocol Items: Recommendations for Interventional Trials (SPIRIT) guidelines^24^ and the SPIRIT-AI extension.^25^ The trial was prospectively registered at ClinicalTrials.gov in June 2025, identifier NCT07034040.

### Eligibility criteria

Potential participants are adults aged 20–59 years living in Norway who have been sick-listed for less than 12 weeks due to back pain, neck pain or widespread pain and who own a smartphone with internet access. The definition of widespread pain was based on the clinical judgement of the referring clinicians. In the study information material, fibromyalgia was given as a typical example.

The following exclusion criteria will be applied: 1) ‘red flags’ indicating possible serious underlying pathology (ie, changes in bladder or bowel function, general feeling of being unwell (malaise), fever and/or unexplained weight loss, reduced muscle (motor) function, sensory loss, walking problems or balance problems, new neck or back pain that started after the age of 55 or feel different from previous pain episodes, pain that does not improve with rest or light activity or pain that is much worse during the night, previous or ongoing cancer, use of steroids/immune-suppressing medications or drug use, pain that is trauma (injury) related) that have not been evaluated by a physician; 2) serious ongoing somatic (eg, unstable heart disease) or mental disorder (eg, psychosis, ongoing manic episode, suicidal ideation) that prevents participation; 3) pain not assessed by a clinician; 4) leg pain that is worse than back pain; 5) unable to take part in exercise/physical activity (eg, use of walking aids, unable to get up and down on the floor independently); 6) current pregnancy; 7) do not have an employer (ie, unemployed or self-employed); 8) insufficient Norwegian language comprehension; 9) sick-listed for more than 3 months in the previous 12 months; 10) a planned return-to-work date within the coming week and 11) previous participation in the trial or concurrent participation in another study that could interfere with the current trial.

### Study context

All legal residents of Norway are covered by the national social insurance system. Medically certified sick leave is covered at 100% for the first 12 months, with some limitations on the size of the salary. The first 16 days are covered by the employer, while the Labor and Welfare Service (Nav) covers the remainder. The employer must, in cooperation with the employee, initiate a follow-up plan before 4 weeks of sick leave and hold a meeting with the sick-listed worker by week 7 of absence. Other stakeholders may be included if relevant. If the employer offers work-related activities or accommodations, the sick-listed worker is expected to participate. Nav has no possibility to sanction the employer if they do not initiate a follow-up plan. Nav is responsible for arranging a meeting with the sick-listed worker and the employer within 26 weeks of sick leave. The sick-listed worker’s general practitioner must attend if requested by the employer or the employee. This is the only mandatory meeting between a sick-listed worker and Nav, and Nav may grant an exemption from holding the meeting. Additional meetings can be held if the sick-listed worker or other stakeholders find it useful.

After 12 months of sick leave, it is possible to apply for long-term medical benefits, work assessment allowance and permanent disability pension, all of which cover about 66% of the original income. Individuals receiving work assessment allowance are expected to work to the extent their work capacity allows.

### Recruitment

**Figure 1:**
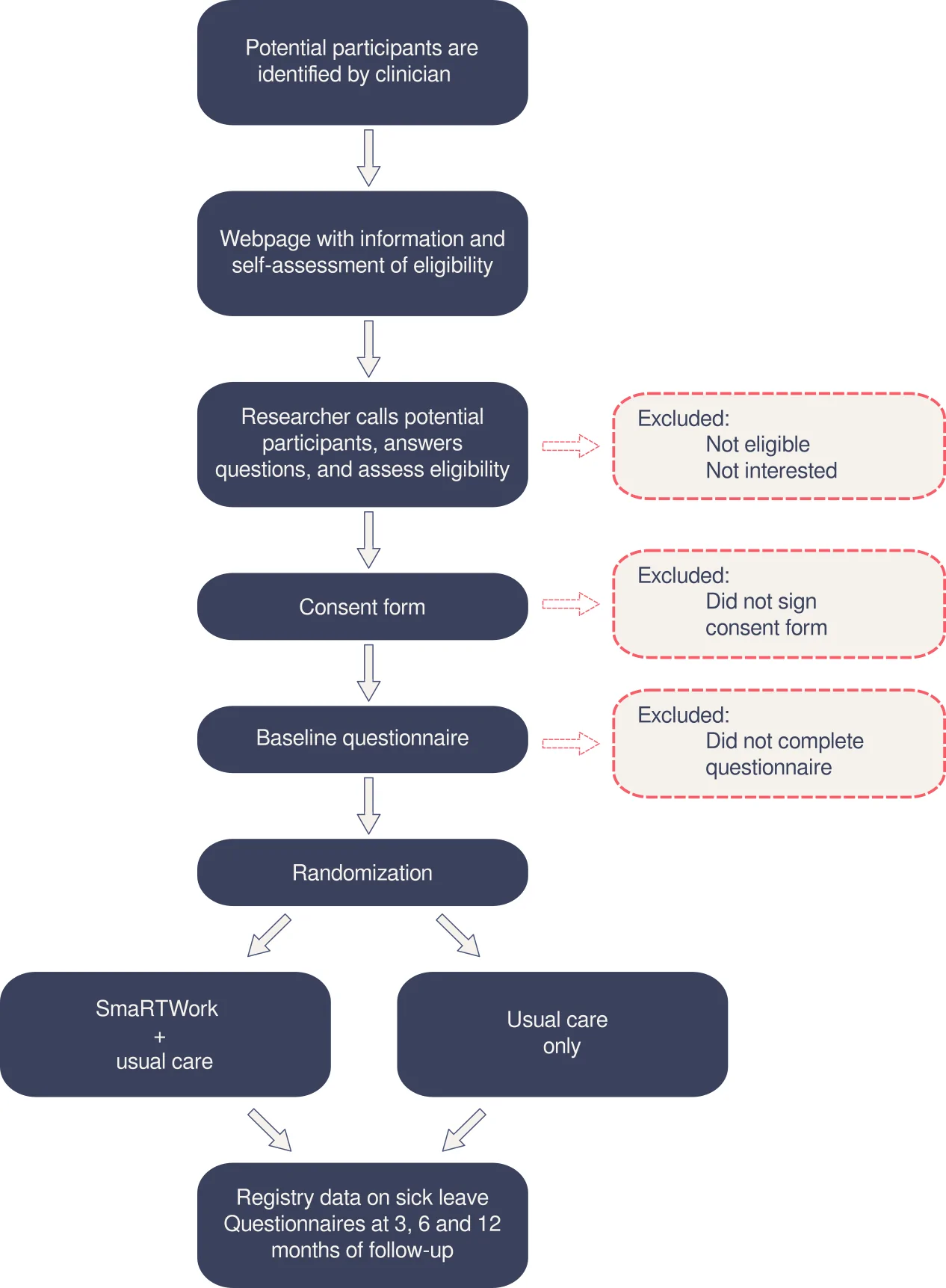
Study procedure and flow of participants through the trial.

Participants are recruited by clinicians that treat MSDs in primary healthcare, that is, general practitioners, chiropractors, physiotherapists and manual therapists. When clinicians identify potential participants based on the inclusion criteria, they provide them with a link or a quick response code to a webpage containing study information and a short screening questionnaire to assess preliminary eligibility (figure 1). Potentially eligible individuals who want to participate submit their contact information to the research team. Final eligibility is assessed by a clinician (physiotherapist, chiropractor or physician) from the research team during a telephone call, which also includes detailed study information and an opportunity to ask questions. If no exclusion criteria apply and the participant wants to continue, they receive a link to the consent form (online supplemental file 3), which is accessed using electronic identification for secure authentication and online signing. Once the consent form is completed, the participant receives an email with a link to the baseline questionnaire. If the questionnaire is not completed within 3 days, a reminder is sent to avoid delays in the enrolment in the trial. A second and final reminder is sent 6 days after the initial email. Participants who consent and complete the questionnaire are randomised to one of the two groups. Figure 1 shows the participant flow. Recruitment for the trial is expected to begin in autumn 2026. Clinicians and participants are recruited nationwide in Norway.

### Randomisation and allocation

Participants are randomised 1:1 to either 1) the SmaRTWork intervention in addition to usual care or 2) usual care only using permuted blocks of random size. The randomisation is performed using a web-based solution (eForsk) provided by the research unit at St Olavs University Hospital, Norway. This unit is a third party and not otherwise involved in the conduct of the trial. To ensure allocation concealment, block sizes are not disclosed to study personnel, and the randomisation code is generated automatically by the software. Participants are informed about their group allocation via email from a project worker not involved in the analyses. Those randomised to the intervention group also receive a link to a webpage with instructions for downloading and using the app, including videos and answers to common technical questions. The app includes an onboarding procedure that explains its core functionality and how to use it.

### Intervention

#### Development of SmaRTWork

**Figure 2:**
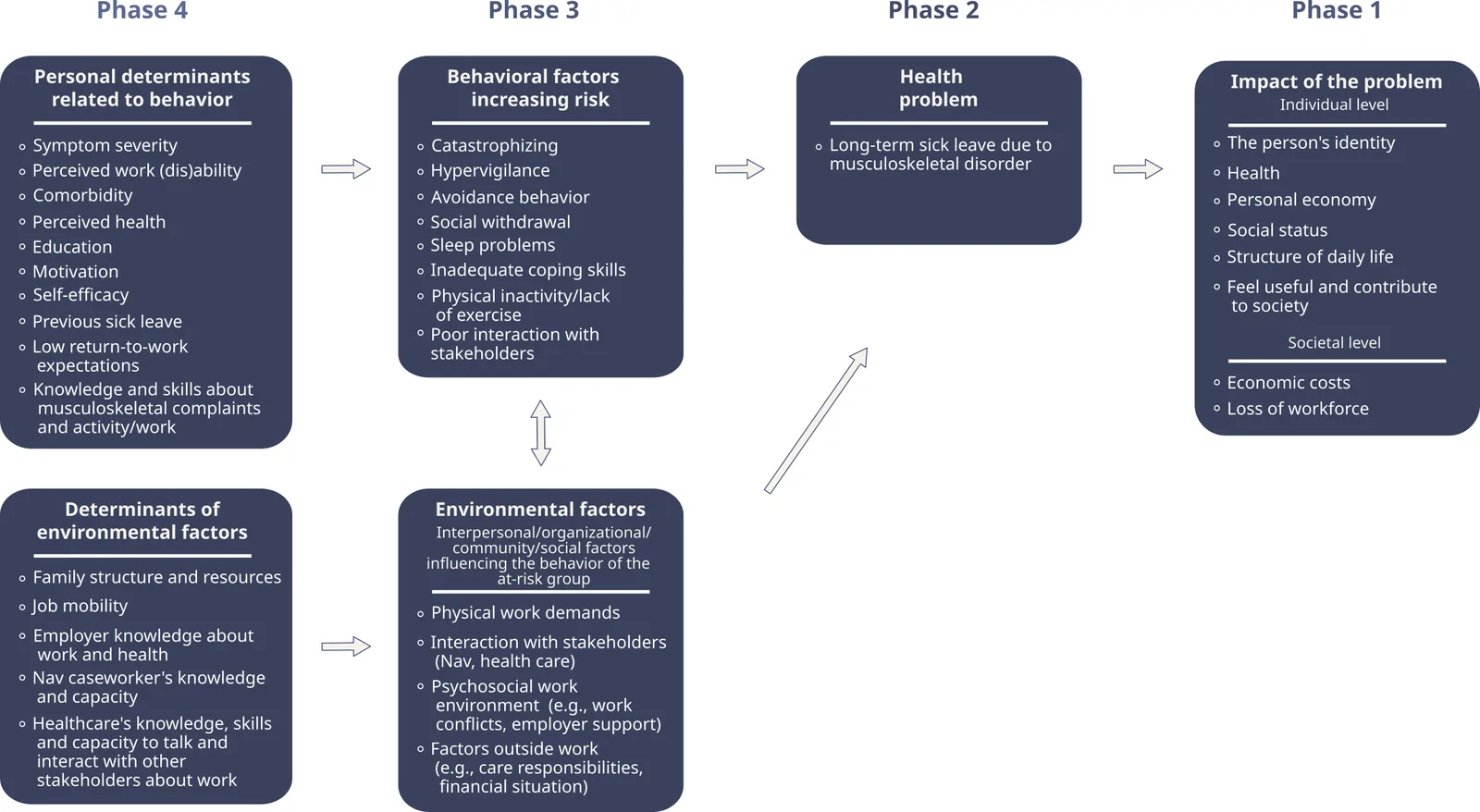
A logic model of the problem based on the PRECEDE model. Nav, Labor and Welfare Service; PRECEDE, Predisposing, Reinforcing, and Enabling Constructs in Educational Diagnosis and Evaluation.

We used the intervention mapping framework^26^ to guide the development of the intervention. This approach ensures that existing evidence, theory, clinical expertise and user and stakeholder perspectives inform all developmental stages of the intervention. The project team involved in the development of the intervention included researchers with backgrounds in medicine (two specialists in physical medicine and rehabilitation and one in insurance medicine), physiotherapy, movement science, computer science, social anthropology, epidemiology, as well as a collaborator from Nav’s research and development department. The research group has experience from several previous trials involving sick-listed workers, and we used knowledge from interviews with sick-listed workers and various stakeholders (Nav caseworkers, clinicians, employers) in previous studies (eg,^27 28^). In addition, we reviewed the literature, performed new interviews with Nav case workers and included expert opinion from clinicians. In step 1 of the intervention mapping process, we developed a logic model of the problem based on the PRECEDE model (figure 2).^29^ The model was developed from right to left but is read from left to right.^26^ The main goal was identified as increasing sustainable return-to-work through strengthening perceived workability. We also identified subgoals: 1) increase self-efficacy for managing musculoskeletal pain and 2) reduce fear and worry about physical activity both in work settings and in everyday life. In step 2, we defined expected programme outcomes and formulated performance objectives for each of the outcomes, that is, a description of specific behaviours the target group must adapt to achieve the desired changes^26^ and steps for how the intervention could target each objective. As this is a self-management intervention, we focused on individual-level and modifiable determinants of return-to-work and MSD management (online supplemental table S1). The last task of step 2 was to create a matrix of change objectives by aligning performance objectives with individual-level determinants, knowledge, skills, self-efficacy and attitudes/beliefs/emotions (online supplemental table S2).

During step 2, it became clear that our objectives aligned closely with the content of the selfBACK app, developed in a recent EU-funded project to support self-management of low back pain.^19 30^ We therefore decided to further develop the selfBACK app for this new target group, with a particular focus on updating and expanding the return-to-work-related educational content. In addition to adding new material, we also conducted interviews with patients (n=12) with MSDs taking part in a rehabilitation programme in secondary care to review and revise the existing content. Technically, the SmaRTWork platform was updated by reimplementing the decision-support backend from the original selfBACK system and modernising the mobile app codebase. The backend was rebuilt to support the SmaRTWork version of the intervention while preserving the core logic needed to deliver individualised self-management recommendations. In parallel, the mobile application was updated to address security vulnerabilities, improve compatibility with current software dependencies and ensure that the app could be maintained and deployed safely in the SmaRTWork study context.

Since the SmaRTWork app is based on the selfBACK app, the theories underpinning behaviour change and engagement remain the same and have been described previously.^31^ In short, the intervention builds on the Transtheoretical Domains Framework,^32^ which is an overall framework encompassing 33 behaviour change theories, and on Normalisation Process Theory,^33^ which helps explain how technology-based interventions are adopted, embedded and integrated into daily routines.^34^

#### The SmaRTWork intervention

Participants randomised to the intervention group will receive access to the SmaRTWork intervention in addition to usual care. The intervention is intended to supplement, not replace, follow-up by healthcare personnel. The intervention consists of an app and a Nav component that put participants in contact with their caseworker at Nav. All participants in the intervention group will get access to the app, while the Nav component will only be offered to those indicating its relevance in the baseline questionnaire.

The app provides individually tailored weekly self-management plans by matching the participant’s health information and sick leave status with tailored educational messages, physical activity advice and exercise recommendations via a decision support system. The system is a data-driven recommender system that uses the case-based reasoning methodology^35 36^ to capture and reuse information from successful participant cases in order to suggest the most suitable self-management plan for a participant (online supplemental figure S1). Case-based reasoning is a well-established methodology within artificial intelligence. Data sources for the case-based reasoning system include participant data collected through the baseline web-based questionnaire and weekly reports submitted by the participant in the SmaRTWork app (including pain, function, fear-avoidance, workability, sick leave status, sleep, self-efficacy, stress, health beliefs and barriers for physical activity). Only questions relevant for a given participant (ie, based on baseline information and progress) are included in the weekly report. This weekly information is used to revise the individually tailored self-management plan by matching the characteristics of the current participant case with existing successful participant cases in the SmaRTWork case-base. A full description of the design of the decision support system has been published elsewhere.^30 37^ All interactions between the participant and the SmaRTWork decision support system happen via the app. There is no interaction between the system and healthcare providers or Nav. The app will be available to the participants in the intervention group the entire duration of the study. Participants decide for themselves how long and how frequently they want to use the app. It is also possible to stop using the app for a period and resume use.

The weekly self-management plan is structured around three main content types: 1) physical activity level and goals (ie, number of steps), 2) strength and flexibility exercises and 3) educational material. Participants are asked to set a physical activity goal, and if they achieved last week’s goal, the app suggests a gradual increase in daily steps. Participants can view their daily step count in the app. The weekly individualised exercise programme includes a short video of each exercise and written instructions. The educational material consists of 21 main components (‘information about musculoskeletal pain’, ‘understanding the mind-body connection’, ‘self-management for musculoskeletal pain’, ‘thoughts, behaviour, attitude and feelings’, ‘fitting in self-management in a busy life’, ‘first aid pain flare-up’, ‘musculoskeletal pain and comorbidities’, ‘goal-setting and action planning’, ‘pacing and graded activity’, ‘problem solving’, ‘relaxation’, ‘sleep and musculoskeletal pain’, ‘social support’, ‘overcoming barriers for self-management of musculoskeletal pain’, ‘keeping active when sick-listed’, ‘staying in contact with the workplace’, ‘handling workplace conflicts’, ‘rights and duties while sick-listed’, ‘work-life interaction’, ‘return-to-work as a process and making a plan’ and ‘considering changing job’). Each day, the participants receive a short educational message (about 140 characters long) tailored to their profile, symptoms and progress. The message also includes a link to a longer, more explanatory text or tools that support self-management of MSDs and return-to-work due to MSDs, such as a goal-setting tool, mindfulness audio and sleep advice.

In addition, the app contains a toolbox and a statistics page for the three components. The toolbox includes a library of educational messages and exercises the participant has received in previous weekly plans, general information on managing MSDs, pain relief exercises that can be used if symptoms worsen and information on sleeping positions when in pain.

To be offered a new self-management plan for the coming week, participants must open the app at least once a week. The app sends push notifications to remind participants to use the app. The goal of the intervention is to help participants acquire the necessary knowledge and skills to self-manage their musculoskeletal complaints and empower them in their return-to-work process. As participants become more confident in their self-management abilities, they may reduce or discontinue app use. Therefore, discontinuation is not necessarily a sign of low compliance but may instead reflect improved self-management. Based on this, per protocol, analyses will not be performed, but we will report on app usage.

The Nav component of the intervention is offered to a subset of participants based on their baseline questionnaire response. There is usually no contact between the sick-listed person and their caseworker at Nav in the initial stage of a sick-leave period. The aim of this component is therefore to establish contact when it is relevant and desired by the sick-listed worker. The baseline questionnaire is designed to identify participants who may face challenges related to i) work accommodations, ii) workplace conflicts, iii) extensive caregiver responsibilities for children, family members or others, iv) financial difficulties or v) concerns about returning to their current workplace. If participants report one or more of these challenges, they are offered to discuss the issue with Nav directly. Participants in the intervention group who indicate that such contact would be useful are called by a research team member, who offers to forward this information to Nav. If the participant agrees, the information is forwarded, and the participant’s caseworker at Nav will reach out to them. Together, the participant and caseworker determine the appropriate measures, which are not restricted by the project.

#### Usual care

Participants randomised to the control group will receive usual care. This consists of sick leave follow-up as described under *Study context* and other recommendations made by the participant’s general practitioner and other clinicians they are seeing, for example, physiotherapists. There will be a considerable variation in these recommendations, as there is no defined treatment for this patient group. Clinical guidelines and recommendations for back, neck and widespread musculoskeletal pain endorse patient education, general physical activity and exercise.^5 38^ Therefore, recommendations by the general practitioner usually include self-management strategies, referrals to physiotherapy or other healthcare professionals or referral for specialist assessment at an outpatient clinic for physical medicine and rehabilitation. The study does not put any restrictions on what is offered as usual care.

### Outcomes

The primary outcome is time to sustainable return-to-work, defined as one consecutive month without receiving medical benefits, assessed over a 12-month period from randomisation using registry data. No individual adjudication will be performed, as the outcome status for each participant will be determined automatically based on an algorithm applied to the registry data. This outcome was selected because the aim of the trial is not only to achieve return-to-work but a sustained return-to-work. This is a widely used outcome in national and international research on return-to-work interventions^21 39 40^ and aligns with ongoing efforts to establish a core outcome set for work participation.^41^ If the intervention is effective, we will also assess long-term outcomes at 2 years and up to 10 years of follow-up. Registry data will be obtained from Nav, which include exact dates of sick leave spells and monthly information on long-term benefits (work assessment allowance and disability pension).

Secondary work participation outcomes include the number of sickness absence days during follow-up and the probability of working (ie, not receiving medical benefits) each month during the 12 months of follow-up, measured as repeated events, based on registry data from Nav. Additional secondary outcomes collected via questionnaires include: musculoskeletal health measured by the Musculoskeletal Health Questionnaire, health-related quality of life measured by the European Quality of Life-5 Dimension questionnaire,^42^ average pain last week measured by a numeric rating scale,^43^ confidence in ability to cope despite pain as assessed with the Pain Self-Efficacy Questionnaire,^44^ depressive symptoms measured by the two-item Patient Health Questionnaire-2,^45^ fear avoidance beliefs measured by the one-item Tampa Scale of Kinesiophobia,^46^ stress measured by the Perceived Stress Scale,^47^ illness perception measured by the Brief Illness Perception Questionnaire,^48^ symptoms of insomnia by items from the HUNT populations study,^49^ work ability measured by the single item from the Work Ability Index^50^ and the overall improvement assessed by the Global Perceived Effect scale.^51^

### Data collection methods and additional data

Questionnaire data are collected by web-based questionnaires at 3, 6 and 12 months of follow-up (online supplemental file 2). Table 1 shows the timeline for the enrolment, interventions and assessments. In addition to the aforementioned questionnaires, the baseline questionnaire includes items on socio-demographic variables: age, gender (man, woman, other gender identity with options non-binary, transgender, intergender, gender questioning, unsure and do not want to answer), height, weight, education (lower secondary school, upper secondary school, vocational school, university/college≤4 years and university/college>4 years), living situation (alone, partner, parents, other adults, young children, older children), work type and employment status, sick leave status, comorbidity (eg, heart disease, diabetes, depression), pain duration, use of pain medication and pain location. The latter is measured through a list with up to nine body parts, such as, neck, back, hips and knees.

**Table 1:** Timeline for enrolment, interventions and assessments

	Enrolment	Allocation	Post-allocation			Tailoring*
Timepoint		Baseline	3 months	6 months	12 months	weekly
**ENROLMENT:**						
Eligibility screen	X					
Informed consent	X					
Allocation		X				
**INTERVENTION/** **COMPARATOR:**						
SmaRTWork		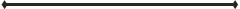				
Usual care		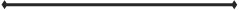				
**ASSESSMENTS:**						
Demographics†		X				
Work type and employment status		X				
Sick leave status		X	X	X	X	X
Factors contributing to sick leave‡		X				
Workability		X	X	X	X	X
Average pain past week		X	X	X	X	X
Worst pain past week		X	X	X	X	
Pain duration		X				
Pain locations		X				
Comorbidity		X				
Health-related quality of life (EQ-5D)		X	X	X	X	
Insomnia symptoms		X	X	X	X	X§
Fear avoidance		X	X	X	X	X§
Pain self-efficacy		X	X	X	X	X§
Brief illness perception		X	X	X	X	
Perceived stress		X	X	X	X	X§
Depressive symptoms		X	X	X	X	X§
Musculoskeletal health		X	X	X	X	
Global perceived effect			X	X	X	
Pain-related function						X
Barriers for self-management						X
Registry data¶		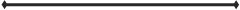				
Qualitative studies					X	

* Weekly responses reported by the participant in the SmaRTWork app. The responses are used to revise the individually tailored self-management plan. Only questions relevant for a given participant (ie, based on baseline information and progress) are included in the weekly reporting.

† Age, gender (man, woman, other gender identity with options non-binary, transgender, intergender, gender questioning, unsure and do not want to answer), height, weight, education (lower secondary school, upper secondary school, vocational school, university/college≤4 years and university/college>4 years), living situation (alone, partner, parents, other adults, young children, older children), work type and employment status.

‡ Challenges related to work accommodations, workplace conflicts, extensive caregiver responsibilities for children, family members or others, financial difficulties and concerns about returning to their current workplace.

§ For the tailoring, only a subset of the items in the questionnaire is used.

¶ Data on use of medical benefits (sick leave, work assessment allowance and disability benefits) and healthcare utilisation (primary and specialist healthcare).

EQ-5D, EuroQol-5 Dimension.

### Qualitative studies and process evaluation

Individual interviews will be conducted with the sick-listed individuals. The interviews will address their experiences with the SmaRTWork app, including their perspectives on individual tailoring, whether the app was user-friendly, their experiences implementing the suggested measures, how the app fit their needs and whether using the app affected their return-to-work process (interview guide in online supplemental file 4). Participants will receive an email invitation to participate in the interviews, including contact information for the researchers. The interviews will be analysed thematically^52^ using a theoretical framework inspired by actor-network theory.^53 54^ Interviews will be performed with participants in the pilot and in the RCT. For the latter group, interviews will be performed after 12 months to avoid influencing the intervention engagement or outcome reporting. We will also interview the case workers involved in delivering the Nav component. Although we aim to conduct focus group interviews with the case workers, we will convert to individual interviews if focus groups are not feasible to arrange (eg, due to participants’ travel time). In addition to the caseworkers’ own experiences, we will use vignettes (ie, anonymised cases) as a background for conversation in these interviews.

Through a process evaluation, facilitators and barriers to implementation will be investigated and described using the Reach, Effectiveness, Adoption, Implementation and Maintenance framework and the Practical Implementation Sustainability Model framework.^55 56^ We will use a mixed-methods design including both quantitative and qualitative methods. Specifically, we will combine quantitative data from the decision support system (eg, app usage, intervention engagement) with data from interviews with the sick-listed workers and case workers at Nav. The findings will contribute to understanding the effect, or lack thereof, of the intervention investigated in the RCT.

### Health economic evaluation

We will perform an economic evaluation, including a cost-effectiveness, a cost-utility and a cost-benefit analysis from both a healthcare and societal perspective. To enable the estimation of individuals’ healthcare costs, information on individuals’ healthcare utilisation will be retrieved from national registries, including the Norwegian Patient Registry and the Municipal Care Registry. Productivity loss due to sick leave will be calculated by multiplying the number of sickness absence days by the average wage in Norway retrieved from Statistics Norway. For the cost-utility analyses, the outcome will be quality-adjusted life years. For the cost-effectiveness analyses, the outcome will be sickness absence days, excluding productivity costs to avoid double counting. Cost-benefit analyses will compare costs and benefits between the intervention and control groups, calculating a net societal benefit. The health economic evaluation will be led by a health economist.

### Sample size

Sample size calculations based on the results of previous return-to-work interventions^57^ indicate that 149 participants are needed in each group (total 298). The calculation is based on a comparison of return-to-work using survival analysis with a log-rank test with a HR of 0.70 (survival probabilities (ie, no return-to-work) 0.1 and 0.2, alpha 0.05, beta 0.20). There will be no loss to follow-up due to the use of registry data for the primary outcome.

A detailed statistical analysis plan will be developed and uploaded to ClinicalTrials.gov before the randomisation code is unblinded. Time to sustainable return-to-work will be described by cumulative probability curves, and group differences will be assessed by HRs from Cox regression. Absolute and relative differences in the 1-year probability of sustainable return-to-work will be estimated from logistic regression, whereas differences in the probability of working each month during follow-up will be estimated using Poisson regression within a generalised estimating equation model. Differences in the number of sickness absence days at specific follow-up times (3, 6, 9 and 12 months) will be estimated using Poisson regression. Mean group differences in continuous secondary outcomes will be analysed using linear mixed models. The precisions of all estimated effects will be assessed by a 95% CI. All analyses will be performed by the intention-to-treat principle. The statistical model assumptions will be evaluated for all analyses, and if these are violated, alternative modelling approaches will be considered. All analyses will be adjusted for factors that are considered predictors of the outcomes under study (eg, education level) to improve precision. The details will be prespecified in the statistical analysis plan.

### Blinding

Due to the nature of the intervention, the participants cannot be blinded to group allocation. The researchers performing analyses and interpretation of the results will be blinded to group allocation. Once the last participant reaches the 12-month follow-up, the data will be extracted from the database for statistical analyses. All personal information that may identify the participants or group allocation will be removed before the blinded analyses, and a blinded interpretation of the main result will be shared within the research group, agreed on and uploaded on ClinicalTrials.gov before group allocation is revealed. The randomisation code (identifying the groups) will be kept in the web solution used for allocation and only available for those performing the allocation.

### Data management and confidentiality

We will comply with the Norwegian University of Science and Technology (NTNU)’s regulations on data storage and data transfer. Data collected by questionnaires, tailoring sessions and the SmaRTWork app will be stored on a password- and firewall-protected server located in the Department of Computer Science at NTNU. Access to the server is restricted to a limited number of researchers involved in the project, and any additional access must be approved by the responsible technical staff. The entire virtual machine is backed up daily, and backups are kept for 1 year. For the RCT analyses, the datasets used will be stored on a secure server at NTNU, where the project manager can grant access to members of the research team. A data protection impact assessment has been completed.

### Adverse effects and trial monitoring

Although serious adverse events are considered unlikely, we have identified several potential risks such as radiculopathy with loss of muscle strength, muscle and tendon ruptures and fractures. Participants are instructed to contact their local healthcare providers as they normally would if their musculoskeletal pain worsens or if they have questions or concerns related to their symptoms. Adverse events, adverse device events, device deficiencies, serious adverse events and serious adverse device events will be monitored weekly during research team meetings. These meetings will also include a review of recruitment, enrolment, data collection, conduct of the intervention and overall progress of the trial. The purpose of this weekly internal audit is to detect any inconsistencies between the planned and actual conduct of the trial and implement corrective measures if needed. If a serious adverse event suspected of being related to the trial occurs, the principal investigator will be responsible for decisions regarding temporary suspension or other necessary actions. Since serious adverse events are not expected, no interim analysis or a priori stopping rules have been defined for this trial. All serious adverse events will be fully recorded by the principal investigator and reported according to Norwegian regulations.

### Protocol amendments

Any amendments to the protocol will be recorded with a detailed description of the change and the implementation date. Any substantial amendments to the protocol related to the SmaRTWork app will be submitted to the Norwegian Medicines Agency for approval before implementation. Any amendment to the protocol that might impact the conduct of the trial will also be submitted to the ethical committee for approval prior to implementation. Furthermore, amendments to the protocol will be registered in the clinical trial registry (www.clinicaltrials.gov) for transparency.

### Patient and public involvement

Three user representatives gave feedback on the different steps of the study, including the questionnaires and the Nav component. Patients with MSDs taking part in rehabilitation gave feedback on the educational content in the app. The patient and public involvement for the development of the selfBACK app has been described previously.^30^

### Research ethics approval and dissemination

The trial is approved by the committees for clinical trials of pharmaceuticals and medical devices in Norway (ref. 563919). Approval of compliance with the relevant national regulations and European Union Guidelines on Medical Software Devices was obtained by the Norwegian Medical Products Agency. All procedures will be in accordance with the ethical standards of the institutional and national research committees and the 1964 Helsinki Declaration and its later amendments.

All eligible participants will receive written information about the study before inclusion, followed by a phone call where they can ask any questions they have about the study. All participants will provide a written (digital) consent prior to enrolment. Participants can also contact the research team at any time during the study period if they have questions or concerns. Only data necessary to answer the study objectives will be collected, thereby minimising the amount of sensitive information. Participants can withdraw from the study at any time-point without giving a reason and without any consequences for their current or future benefits. For participants who are put in contact with Nav, the type of benefit may change if the participant and their caseworker determine that it is appropriate.

We will publish the trial results in international peer-reviewed journals. Results will also be presented at relevant national and international conferences and to the public.

## Supplementary material

10.1136/bmjopen-2026-122680 online supplemental file 1

10.1136/bmjopen-2026-122680 online supplemental file 2

10.1136/bmjopen-2026-122680 online supplemental file 3

10.1136/bmjopen-2026-122680 online supplemental file 4
