# Supplementary material for: Effectiveness of an artificial intelligence-based self-management intervention to support sick-listed individuals with musculoskeletal disorders in Norway: rationale and protocol for the SmaRTWork randomised controlled trial
Source: BMJ Open. 2026 Aug 18;16(8):e122680. doi: 10.1136/bmjopen-2026-122680 (PMC13504858; doi:10.1136/bmjopen-2026-122680)
Supplement: Supplementary file 1 [file bmjopen-16-8-s001.pdf]

Online supplementary

Table S1 Expected program outcomes, performance objectives, and practical steps for the interventions to target them.

**Behavioral outcome (what needs to change)**	**Associated performance objective (description of the specific behaviors the at-risk group has to perform to achieve the desired change)**	**Practical step**
Reduced fear of movement	Changes in pain beliefs (e.g., reduce catastrophizing and kinesiophobia)	Education (reassurance, knowledge about pain, no tissue damage etc)<br>Exercises
	Are physically active despite some pain	Education<br>Exercises<br>Steps
	Do normal daily activities despite (fear of) pain	Education<br>Exercises
Reduced hypervigilance	Changes in pain beliefs	Education<br>Mindfullness
Reduce passive and/or overly active behaviors	Learn to balance activity and rest	Education<br>Mindfullness
Avoid isolation	Engage in valued social activities and outside activities	Education
Sleep better	Use general sleep advice if sleeping problems	Education,<br>Sleep tool
	Are physically active	Education<br>Exercises<br>Steps
Understand the relation between mood and pain	Increased knowledge on mood and pain	Education
Adjusting work demands	Address need for accommodations for heavy work or other work demands necessary.	Education<br>Nav component
Reduce work conflict	Address work conflicts	Education<br>Nav component
Understand the impact of factors outside work on workability	Address factors outside affecting work (that are possible to adjust, e.g., financial problems)	Education<br>Nav component
Have a plan for return-to-work	Create a return-to-work plan	Education

Table S2 Change objectives of the intervention (the cells in the matrix) based on performance objectives and personal determinants.

**Performance objective** (description of the specific behaviors the at-risk group have to perform to achieve the desired change)	**Personal determinants**			
	**Knowledge**	**Skills**	**Self-efficacy**	**Attitudes/beliefs /emotions**
Changes in pain beliefs	Understand the difference between pain and harm	Ability to cope with pain	Avoids passive coping	Feel safe being active and resume work. Not fearful of re-injury/aggravation
Are active despite pain/fear of pain	Understand the difference between pain and harm, and acute and chronic pain	Ability to cope with pain associated with activity	Believe can be active despite pain. Avoid passive coping	Feel safe being active and resume work. Not fearful of re-injury/aggravation
Balance activity and rest	Understands the need for balance between activity and rest. Understanding of graded activity as a tool to increase function	Ability to adapt activity levels	Believe an active life is possible, believe can manage the balance of activity and rest	Not fearful of activity, and acceptance of the need for balance in activity levels
Are physically active	Understand that activity is beneficial for physical and mental health	Ability to plan for some physical activity each day	Believe can find different types of physical activity that are possible to perform	Feel safe being physically active
Engages in valued social activities and outside activities	Understand the importance of activity outside the home and social interactions	Cope with others having other opinions about being active despite on sick leave	Believe can be active and social despite sick leave	Less worry what others think about being active and social despite being on sick leave

Increased knowledge on mood and pain	Understanding the interaction between mental health and pain.	Understand how to cope with mood changes due to pain and what to do if there is a need for professional help	Believe can cope with effect of pain on mood and if necessary, who to contact for help	Less worry about mood changes and openness for mental health treatment if necessary
Use general sleep advice (if sleeping problems)	Knowledge about general sleep advice	Cope with sleep problems	Believe can cope with sleep problems and improve sleep	Less worry about sleep problems
Address factors outside affecting work (that are possible to adjust, e.g., financial problems)	Understand that stress in private life affect pain and workability	Know who to contact to discuss problems and for support in addressing problem	Believe problems can be solved or managed in another way	Less stress
Address work conflicts	Understand that the conflict is important for sick leave and needs to be addressed	Know who to contact for support in addressing problem	Believe problem can be solved or that can find new work	Less stress
Address need for accommodations for heavy work or other work demands necessary.	Understand that the work demands should be addressed to help RTW process	Ability to contact employer about situation or know who to contact for support	Believe work demands can be addressed	Open for accommodations in work tasks might be necessary

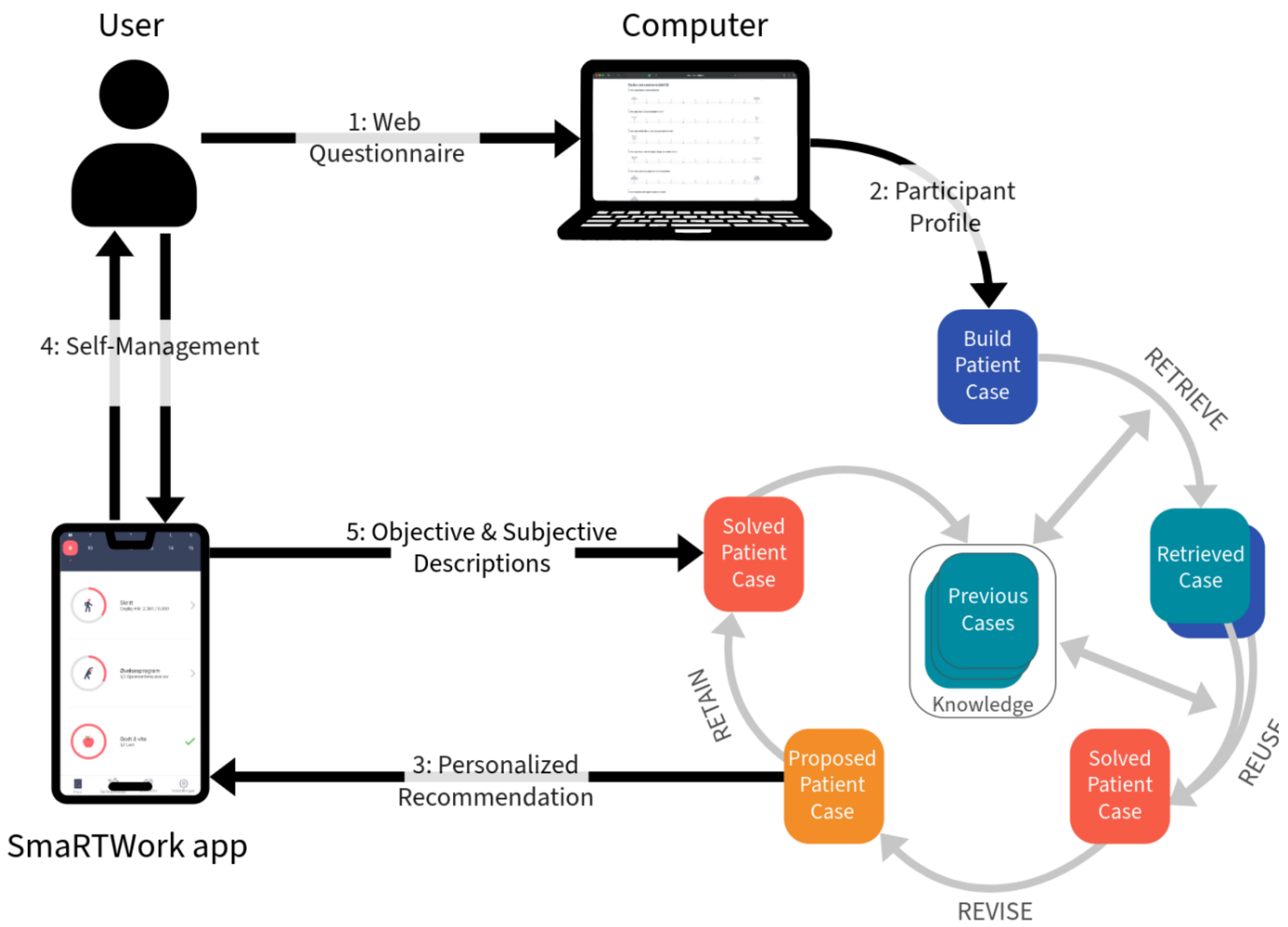

Figure S1 Overview of the core components of the SmaRTWork system and the user interaction with the different components. The case-based reasoning cycle is shown to the right. (**1**) Based on data from the baseline questionnaire, a participant risk profile is created. (**2**). This current user profile (=patient case) is then compared to previous similar successful profiles (cases) available in the knowledge base of the case-based reasoning system. The case in the knowledge base that best matches the current case is reused and adapted to recommend measures for the current participant (**3**). The participant will receive the recommendations through a weekly plan in the SmaRTWork app (**4**). The weekly self-management plan is structured around a) physical activity level (i.e., number of steps) and goals, b) strength and flexibility exercises and c) educational material. On a weekly basis, the participant reports back to the system via an automated Q/A session, including pain, function, fear-avoidance, workability, sick leave status, sleep, self-efficacy, stress, health beliefs, and barriers for physical activity (**7**). This information is fed back to the case-based reasoning system, and the sick-listed worker receives updated recommendations.
