# Supplementary material for: Effectiveness of an artificial intelligence-based self-management intervention to support sick-listed individuals with musculoskeletal disorders in Norway: rationale and protocol for the SmaRTWork randomised controlled trial
Source: BMJ Open. 2026 Aug 18;16(8):e122680. doi: 10.1136/bmjopen-2026-122680 (PMC13504858; doi:10.1136/bmjopen-2026-122680)
Supplement: Supplementary file 2 [file bmjopen-16-8-s002.pdf]

SmaRTWork

Det er 101 spørsmål i undersøkelsen.

NTNU | Kunnskap for en bedre verden

Takk for at du ønsker å delta i forskningsprosjektet SmaRTWork! Vi setter pris på bidraget ditt, det vil kunne hjelpe deg og andre som er sykmeldt på grunn av muskel- og skjelettsmerter.

For å kunne sammenligne resultatene med andre studier har vi hentet spørsmål fra tidligere forskningsprosjekter. Spørsmålene handler om helsen og arbeidssituasjonen din. Selv om mange av spørsmålene ligner på hverandre, er de ikke helt identiske. Derfor ønsker vi at du svarer på alle spørsmålene så godt du kan. Hvis du føler at flere eller ingen av svaralternativene passer, velger du det du tenker er mest riktig. Det er ikke meningen at du skal bruke mye tid på hvert spørsmål. Svar det første som faller deg inn.

Det tar ca. 10-15 minutter å svare på spørreskjemaet. Svarene dine vil bli behandlet konfidensielt.

Vennligst trykk på ’Neste’ for å starte utfyllingen.

Svar kun på dette hvis følgende betingelser er oppfylt:
TOKEN:ATTRIBUTE_1 == 'baseline'

NTNU | Kunnskap for en bedre verden

Tusen takk for at du deltar i SmaRTWork studien.

Vi ønsker å vite hvordan det går med deg 3 måneder etter oppstart i studien. Dette er et oppfølgingsspørreskjema som det tar ca. 10 minutter å svare på.

Trykk på ’Neste’ for å starte utfyllingen.

Svar kun på dette hvis følgende betingelser er oppfylt:
TOKEN:ATTRIBUTE_1 == 'followup1'

NTNU | Kunnskap for en bedre verden

Tusen takk for at du deltar i SmaRTWork studien.

Vi ønsker å vite hvordan det går med deg 6 måneder etter oppstart i studien. Dette er et oppfølgingsspørreskjema som det tar ca. 10 minutter å svare på.

Trykk på 'Neste' for å starte utfyllingen.

Svar kun på dette hvis følgende betingelser er oppfylt:
TOKEN:ATTRIBUTE_1 == 'followup2'

NTNU | Kunnskap for en bedre verden

Tusen takk for at du deltar i SmaRTWork studien.

Vi ønsker å vite hvordan det går med deg 12 måneder etter oppstart i studien. Dette er et oppfølgingsspørreskjema som det tar ca. 10 minutter å svare på.

Trykk på 'Neste' for å starte utfyllingen.

Svar kun på dette hvis følgende betingelser er oppfylt:
TOKEN:ATTRIBUTE_1 == 'followup3'

Personlig profil

Hvor gammel er du? *

Svaret ditt må være mellom 18 og 99
Dette feltet kan bare inneholde heltallsverdier.
Vennligst skriv her:

Hvilket kjønn identifiserer du deg som? *

Velg kun en av følgende:

- Mann
- Kvinne
- Annen kjønnsidentitet
- Ønsker ikke å svare

Hva identifiserer du deg som? *

Svar kun på dette hvis følgende betingelser er oppfylt:
Svaret var 'Annen kjønnsidentitet ' ved spørsmål ' [Gender]' (Hvilket kjønn identifiserer du deg som?)

Velg ett av alternativene
Velg kun en av følgende:

- Ikke-binær
- Transperson
- Interkjønn
- Søkende
- Usikker
- Ønsker ikke å svare

Kroppshøyde *

Svaret ditt må være mellom 121 og 215
Vennligst skriv her:

Kroppsvekt *

Svaret ditt må være mellom 35 og 300
Vennligst skriv her:

kg

*

Svar kun på dette hvis følgende betingelser er oppfylt:
! is_empty(Height) and ! is_empty(Weight)

Familie

Bor du alene eller sammen med andre? *

Velg kun en av følgende:

- Alene
- Sammen med andre

Hvem bor du sammen med? (flere kryss er mulig) *

Svar kun på dette hvis følgende betingelser er oppfylt:
LiveAlone == "with"

Vennligst velg alle som passer:

- [ ] Partner eller ektefelle
- [ ] Foreldre eller svigerforeldre
- [ ] Andre voksne
- [ ] Barn under 5 år
- [ ] Barn 6 - 15 år
- [ ] Barn over 15 år

Utdanning

Hva er din høyeste fullførte utdanning? *

Velg kun en av følgende:

- ○ Ungdomsskole
- ○ Videregående skole
- ○ Fagbrev/fagutdanning
- ○ Inntil 4 år høyskole/universitet
- ○ Mer enn 4 år høyskole/universitet

Nåværende arbeidsforhold

Har du for øyeblikket arbeid/ansettelse? (kun ett kryss mulig)
*

Velg kun en av følgende:

- Ja, fulltidsjobb
- Ja, deltidsjobb
- Nei, ikke jobb: arbeidsledig eller permittert
- Nei, ikke jobb: annen årsak

Ikke ta hensyn til at du er sykmeldt.

Hvor stor stillingsandel har du?

*

Svar kun på dette hvis følgende betingelser er oppfylt:
Svaret var 'Ja, deltidsjobb' ved spørsmål ' [Employment]' (Har du for øyeblikket arbeid/ansettelse? (kun ett kryss mulig))

Velg kun en av følgende:

- 1 til 24%
- 25-49%
- 50-74%
- 75-99%
- 100% eller mere

Ikke ta hensyn til at du er sykmeldt. Har du flere arbeidsforhold så krysser du av for den samlede andelen

Beskrivelse av arbeidet ditt

Hvis du er i lønnet arbeid, hvordan vil du **beskrive arbeidet ditt**?

Hvis flere av alternativene passer velger du det du føler **best** reflekterer din arbeidshverdag (sett ett kryss) *

Velg kun en av følgende:

- ◯ For det meste stillesittende (for eksempel kontorarbeid)
- ◯ Arbeid som krever at du går eller står mye (for eksempel lett industriarbeid, rengjøring)
- ◯ Arbeid hvor du går og løfter mye (for eksempel postbud, sykepleier, bygningsarbeid)
- ◯ Tungt kroppsarbeid (for eksempel skogsarbeider, tungt jordbruksarbeid)

Smerter siste uke

Hvor sterke smerter har du hatt i **gjennomsnitt** den siste uken på en skala fra 0 (ingen smerter) til 10 (verst tenkelige smerter)? *

Vennligst velg passende besvarelse til hvert alternativ:

	Ingen smerter 0	1	2	3	4	5	6	7	8	9	Verst tenkelig smerter 10
**Smerter i gjennomsnitt**	◯	◯	◯	◯	◯	◯	◯	◯	◯	◯	◯

Hvor sterke har de **sterkeste** smertene dine vært den siste uken på en skala fra 0 (ingen smerter) til 10 (verst tenkelige smerter)? *

Vennligst velg passende besvarelse til hvert alternativ:

	Ingen smerter 0	1	2	3	4	5	6	7	8	9	Verst tenkelig smerter 10
**De sterkeste smertene**	○	○	○	○	○	○	○	○	○	○	○

Hvor mange dager i løpet av den siste uken har du brukt smertestillende medisiner for smertene dine? *

Velg ett av alternativene
Velg kun en av følgende:

- ○ Daglig
- ○ 3-6 dager
- ○ 1-2 dager
- ○ Ingen

Smertevarighet

Hvor lenge har du hatt de nåværende smertene?
*

Svar kun på dette hvis følgende betingelser er oppfylt:
(((! is_empty(PainIntensityAver_average.NAOK) && (PainIntensityAver_average.NAOK == 1)) or (! is_empty(PainIntensityAver_average.NAOK) && (PainIntensityAver_average.NAOK == 10)) or (! is_empty(PainIntensityAver_average.NAOK) && (PainIntensityAver_average.NAOK == 2)) or (! is_empty(PainIntensityAver_average.NAOK) && (PainIntensityAver_average.NAOK == 3)) or (! is_empty(PainIntensityAver_average.NAOK) && (PainIntensityAver_average.NAOK == 4)) or (! is_empty(PainIntensityAver_average.NAOK) && (PainIntensityAver_average.NAOK == 5)) or (! is_empty(PainIntensityAver_average.NAOK) && (PainIntensityAver_average.NAOK == 6)) or (! is_empty(PainIntensityAver_average.NAOK) && (PainIntensityAver_average.NAOK == 7)) or (! is_empty(PainIntensityAver_average.NAOK) && (PainIntensityAver_average.NAOK == 8)) or (! is_empty(PainIntensityAver_average.NAOK) && (PainIntensityAver_average.NAOK == 9))) or ((! is_empty(PainIntensityWors_worst.NAOK) && (PainIntensityWors_worst.NAOK == 1)) or (! is_empty(PainIntensityWors_worst.NAOK) && (PainIntensityWors_worst.NAOK == 10)) or (! is_empty(PainIntensityWors_worst.NAOK) && (PainIntensityWors_worst.NAOK == 2)) or (! is_empty(PainIntensityWors_worst.NAOK) && (PainIntensityWors_worst.NAOK == 3)) or (! is_empty(PainIntensityWors_worst.NAOK) && (PainIntensityWors_worst.NAOK == 4)) or (! is_empty(PainIntensityWors_worst.NAOK) && (PainIntensityWors_worst.NAOK == 5)) or (! is_empty(PainIntensityWors_worst.NAOK) && (PainIntensityWors_worst.NAOK == 6)) or (! is_empty(PainIntensityWors_worst.NAOK) && (PainIntensityWors_worst.NAOK == 7)) or (! is_empty(PainIntensityWors_worst.NAOK) && (PainIntensityWors_worst.NAOK == 8)) or (! is_empty(PainIntensityWors_worst.NAOK) && (PainIntensityWors_worst.NAOK == 9))))

Velg kun en av følgende:

- ◯ Mindre enn 4 uker
- ◯ 5 til 12 uker
- ◯ 3 til 6 måneder
- ◯ 7 til 12 måneder
- ◯ Lengre enn 12 måneder

Smertelokalisering

Kryss av for de områdene der du for tiden har smerter (flere kryss er mulig) *

Svar kun på dette hvis følgende betingelser er oppfylt:
(((! is_empty(PainIntensityAver_average.NAOK) && (PainIntensityAver_average.NAOK == 1)) or (! is_empty(PainIntensityAver_average.NAOK) && (PainIntensityAver_average.NAOK == 10)) or (! is_empty(PainIntensityAver_average.NAOK) && (PainIntensityAver_average.NAOK == 2)) or (! is_empty(PainIntensityAver_average.NAOK) && (PainIntensityAver_average.NAOK == 3)) or (! is_empty(PainIntensityAver_average.NAOK) && (PainIntensityAver_average.NAOK == 4)) or (! is_empty(PainIntensityAver_average.NAOK) && (PainIntensityAver_average.NAOK == 5)) or (! is_empty(PainIntensityAver_average.NAOK) && (PainIntensityAver_average.NAOK == 6)) or (! is_empty(PainIntensityAver_average.NAOK) && (PainIntensityAver_average.NAOK == 7)) or (! is_empty(PainIntensityAver_average.NAOK) && (PainIntensityAver_average.NAOK == 8)) or (! is_empty(PainIntensityAver_average.NAOK) && (PainIntensityAver_average.NAOK == 9))) or ((! is_empty(PainIntensityWors_worst.NAOK) && (PainIntensityWors_worst.NAOK == 1)) or (! is_empty(PainIntensityWors_worst.NAOK) && (PainIntensityWors_worst.NAOK == 10)) or (! is_empty(PainIntensityWors_worst.NAOK) && (PainIntensityWors_worst.NAOK == 2)) or (! is_empty(PainIntensityWors_worst.NAOK) && (PainIntensityWors_worst.NAOK == 3)) or (! is_empty(PainIntensityWors_worst.NAOK) && (PainIntensityWors_worst.NAOK == 4)) or (! is_empty(PainIntensityWors_worst.NAOK) && (PainIntensityWors_worst.NAOK == 5)) or (! is_empty(PainIntensityWors_worst.NAOK) && (PainIntensityWors_worst.NAOK == 6)) or (! is_empty(PainIntensityWors_worst.NAOK) && (PainIntensityWors_worst.NAOK == 7)) or (! is_empty(PainIntensityWors_worst.NAOK) && (PainIntensityWors_worst.NAOK == 8)) or (! is_empty(PainIntensityWors_worst.NAOK) && (PainIntensityWors_worst.NAOK == 9))))

Vennligst velg alle som passer:

- ☐ Nakke
- ☐ Skuldre
- ☐ Øvre del av ryggen
- ☐ Albuer
- ☐ Korsryggen
- ☐ Håndledd/hender

- [ ] Hofter/lår
- [ ] Knær
- [ ] Ankler/føtter
- [ ] Ingen av ovennevnte

*

Sykefravær

Hvilken sykmeldingsgrad har du for tiden?
*

Velg kun en av følgende:

- ◯ Fullt sykmeldt
- ◯ Delvis sykmeldt
- ◯ Jeg er ikke sykmeldt

Har du vært borte fra jobben på grunn av smerter **før** den nåværende sykmeldingen?
*

Svar kun på dette hvis følgende betingelser er oppfylt:
TOKEN:ATTRIBUTE_1 == 'baseline'

Velg kun en av følgende:

- ◯ Nei
- ◯ Ja, men ikke sykmeldt
- ◯ Ja, har vært sykmeldt tidligere på grunn av smerter

Hvor lenge tror du at du vil være sykmeldt fra i dag?
*

Svar kun på dette hvis følgende betingelser er oppfylt:
Svaret var 'Delvis sykmeldt' *eller* 'Fullt sykmeldt' ved spørsmål ' [SickLeaveGrade]' (Hvilken sykmeldingsgrad har du for tiden?)

Velg kun en av følgende:

- Mindre enn 4 uker
- 5 til 12 uker
- 3 til 6 måneder
- 7 til 12 måneder
- Lengre enn 12 måneder

Arbeidsevne

Vurder din nåværende arbeidsevne sammenlignet med da den var på sitt beste. Arbeidsevnen på sitt beste verdsettes med 10 poeng.
*

Vennligst velg passende besvarelse til hvert alternativ:

Helt uten arbeids-evne 0	1	2	3	4	5	6	7	8	9	Arbeidsev på sitt beste 10
○	○	○	○	○	○	○	○	○	○	○

Kontakt med Nav

Hvilken kontakt har du hatt med Nav mens du har vært med i denne studien (flere kryss er mulig)? *

Velg alt som passer

Vennligst velg alle som passer:

- ☐ Kun kontakt i forbindelse med utfylling av sykemeldingen
- ☐ Økonomisk støtte/rådgivning (se bort fra støtte i form av sykepenger)
- ☐ Tilrettelegging og/eller hjelpemidler på arbeidsplassen
- ☐ Arbeidsrettet tiltak (som for eksempel arbeidstrening, kurs eller utdanning)
- ☐ Hjelp til jobbskifte
- ☐ Håndtering av konflikt på arbeidsplassen
- ☐ Dialogmøte med Nav, arbeidsgiver og evt fastlege
- ☐ Annet
- ☐ Ingen ting

Tilpasninger på arbeidsplassen

Tror du tilpasninger på arbeidsplassen (f.eks andre arbeidsoppgaver, endrede vakter) ville gjort det lettere for deg å komme tilbake i jobb?

*

Velg kun en av følgende:

- ◯ Ja
- ◯ Nei
- ◯ Vet ikke

Hvordan er mulighetene for tilpasninger på arbeidsplassen din?
*

Svar kun på dette hvis følgende betingelser er oppfylt:
AdaptWork1 == "Y" or AdaptWork1 == "notk"

Velg kun en av følgende:

- ◯ Det er vanskelig å få gjort tilpasninger der jeg jobber
- ◯ Tilpasninger har ikke vært et tema
- ◯ Det er gjort tilpasninger

Tror du det ville vært nyttig for deg å få kontakt med din Nav veileder for å diskutere mulige tilpasninger på arbeidsplassen din? *

Svar kun på dette hvis følgende betingelser er oppfylt:
Svaret var 'Det er vanskelig å få gjort tilpasninger der jeg jobber' *eller* 'Tilpasninger har ikke vært et tema' ved spørsmål ' [AdaptWork2]' (Hvordan er mulighetene for tilpasninger på arbeidsplassen din?)

Velg kun en av følgende:

- ◯ Ja
- ◯ Nei
- ◯ Jeg er allerede i kontakt med min Nav veileder på grunn av denne problemstillingen

Konflikter på arbeidsplassen

Har du opplevd konflikter eller lignende ubehag på arbeidsplassen din?
*

Velg kun en av følgende:

- Ja, det er medvirkende til at jeg er sykmeldt
- Ja, men det har ikke påvirket at jeg ble sykmeldt
- Ja tidligere, men er ikke et problem lengere
- Nei

Tror du det ville vært nyttig for deg å få kontakt med din Nav veileder for å diskutere arbeidssituasjonen din?
*

Svar kun på dette hvis følgende betingelser er oppfylt:
Svaret var 'Ja, det er medvirkende til at jeg er sykmeldt' ved spørsmål ' [ConflWork1]' (Har du opplevd konflikter eller lignende ubehag på arbeidsplassen din?)

Velg kun en av følgende:

- Ja
- Nei
- Jeg er allerede i kontakt med min NAV veileder på grunn av denne problemstillingen

Arbeidssituasjon

Er det sannsynlig at du kommer tilbake til jobben du har i dag?
*

Velg kun en av følgende:

- Ja
- Nei
- Vet ikke

Tror du det ville vært nyttig for deg å få kontakt med din Nav veileder for å diskutere andre jobbmuligheter?
*

Svar kun på dette hvis følgende betingelser er oppfylt:
Svaret var 'Nei' *eller* 'Vet ikke' ved spørsmål ' [WorkSatisfaction]' (Er det sannsynlig at du kommer tilbake til jobben du har i dag?)

Velg kun en av følgende:

- Ja
- Nei
- Jeg er allerede i kontakt med min Nav veileder på grunn av denne problemstillingen

Belastninger utenfor jobb

Jeg har store omsorgsoppgaver for barn
*

Velg kun en av følgende:

- Ja
- Nei

Jeg har store omsorgsoppgaver for familie/andre *

Velg kun en av følgende:

- Ja
- Nei

Jeg har økonomiske utfordringer *

Velg kun en av følgende:

- Ja
- Nei

Tror du noen av belastningene du opplever påvirker arbeidsevnen din? *

Svar kun på dette hvis følgende betingelser er oppfylt:
((StrainOutWorkchild.NAOK == "Y") or (StrainOutWorkCar.NAOK == "Y") or (StrainOutWorkEco.NAOK == "Y"))

Velg kun en av følgende:

- Ja
- Kanskje
- Nei

Tror du det ville vært nyttig for deg å få kontakt med din Nav veileder for å diskutere belastningene utenfor jobb?
*

Svar kun på dette hvis følgende betingelser er oppfylt:
Svaret var 'Kanskje' *eller* 'Ja' ved spørsmål ' [StrainOutWork2]' (Tror du noen av belastningene du opplever påvirker arbeidsevnen din?)

Velg kun en av følgende:

- Ja
- Nei
- Jeg er allerede i kontakt med min Nav veileder på grunn av denne problemstillingen

Mosjon og trening

Hvor ofte mosjonerer du? (ta et gjennomsnitt)

Med mosjon mener vi at du f.eks. går tur, går på ski, sykler, svømmer eller driver trening/idrett. *

Velg kun en av følgende:

- Aldri
- Sjeldnere enn en gang i uka
- En gang i uka
- 2-3 ganger i uka
- Omtrent hver dag

Dersom du driver slik mosjon så ofte som en eller flere ganger i uka; hvor hardt mosjonerer du? (Ta et gjennomsnitt)

*

Svar kun på dette hvis følgende betingelser er oppfylt:
Svaret var '2-3 ganger i uka' *eller* 'Omtrent hver dag' *eller* 'En gang i uka' ved spørsmål ' [Hunt4a]' (Hvor ofte mosjonerer du? (ta et gjennomsnitt) Med mosjon mener vi at du f.eks. går tur, går på ski, sykler, svømmer eller driver trening/idrett.)

Velg kun en av følgende:

- ◯ Tar det rolig uten å bli andpusten eller svett
- ◯ Tar det så hardt at jeg blir andpusten eller svett
- ◯ Tar meg nesten helt ut

Hvor lenge holder du på hver gang? (Ta et gjennomsnitt)

*

Svar kun på dette hvis følgende betingelser er oppfylt:
Svaret var 'Sjeldnere enn en gang i uka' *eller* 'Omtrent hver dag' *eller* '2-3 ganger i uka' *eller* 'En gang i uka' ved spørsmål ' [Hunt4a]' (Hvor ofte mosjonerer du? (ta et gjennomsnitt) Med mosjon mener vi at du f.eks. går tur, går på ski, sykler, svømmer eller driver trening/idrett.)

Velg kun en av følgende:

- ◯ Mindre enn 15 minutter
- ◯ 15-29 minutter
- ◯ 30-60 minutter
- ◯ Mer enn 60 minutter

Andre sykdommer og plager

Har du, eller har du noen gang hatt, noen av disse sykdommene/plagene? (flere kryss er mulig) *

Vennligst velg alle som passer:

- [ ] Hjerte- og karsykdom (hjerteinfarkt, angina pectoris [hjertekrampe], andre hjerte- og karsykdommer)
- [ ] Hjertesvikt
- [ ] Hjerneslag/hjerneblødning
- [ ] Astma
- [ ] Kronisk bronkitt, emfysem, KOLS
- [ ] Diabetes (sukkersyke)
- [ ] Mage- og tarmsykdommer (Crohn's sykdom, ulcerøs kolitt, cøliaki, eller lignende)
- [ ] Nyresykdom
- [ ] Kreftsykdom
- [ ] Epilepsi
- [ ] Beinskjørhet (osteoporose)
- [ ] Slitasjegikt (artrose)
- [ ] Depresjon som påvirker hverdagen din
- [ ] Angst som påvirker hverdagen din
- [ ] Søvnapné, narkolepsi eller andre søvnsykdommer
- [ ] Leddgikt (reumatoid artritt)
- [ ] Psoriasis artritt/psoriasis
- [ ] Andre kroniske sykdommer
- [ ] Ingen sykdommer/plager

Generell helse og livskvalitet

Vennligst kryss av den ENE boksen som best beskriver helsen din I DAG.

MOBILITET *

Velg kun en av følgende:

- Jeg har ingen problemer med å gå omkring.
- Jeg har litt problemer med å gå omkring.
- Jeg har middels store problemer med å gå omkring.
- Jeg har store problemer med å gå omkring.
- Jeg er ute av stand til å gå omkring.

© EuroQol Research Foundation. EQ-5D™ is a trade mark of the EuroQol Research Foundation. UK (English) v2.0

*

© EuroQol Research Foundation. EQ-5D™ is a trade mark of the EuroQol Research Foundation

Svar kun på dette hvis følgende betingelser er oppfylt:
0

Generell helse og livskvalitet

Vennligst kryss av den ENE boksen som best beskriver helsen din I DAG.

PERSONLIG STELL *

Velg kun en av følgende:

- ◯ Jeg har ingen problemer med å vaske meg eller kle meg.
- ◯ Jeg har litt problemer med å vaske meg eller kle meg.
- ◯ Jeg har middels store problemer med å vaske meg eller kle meg.
- ◯ Jeg har store problemer med å vaske meg eller kle meg.
- ◯ Jeg er ute av stand til å vaske meg eller kle meg.

© EuroQol Research Foundation. EQ-5D™ is a trade mark of the EuroQol Research Foundation. UK (English) v2.0

*

© EuroQol Research Foundation. EQ-5D™ is a trade mark of the EuroQol Research Foundation

Svar kun på dette hvis følgende betingelser er oppfylt:
0

Generell helse og livskvalitet

Vennligst kryss av den ENE boksen som best beskriver helsen din I DAG.

VANLIGE GJØREMÅL (f.eks. arbeid, studier, husarbeid, familie- eller fritidsaktiviteter) *

Velg kun en av følgende:

- ◯ Jeg har ingen problemer med å utføre mine vanlige gjøremål.
- ◯ Jeg har litt problemer med å utføre mine vanlige gjøremål.
- ◯ Jeg har middels store problemer med å utføre mine vanlige gjøremål.
- ◯ Jeg har store problemer med å utføre mine vanlige gjøremål.
- ◯ Jeg er ute av stand til å utføre mine vanlige gjøremål.

© EuroQol Research Foundation. EQ-5D™ is a trade mark of the EuroQol Research Foundation. UK (English) v2.0

*

© EuroQol Research Foundation. EQ-5D™ is a trade mark of the EuroQol Research Foundation

Svar kun på dette hvis følgende betingelser er oppfylt:
0

Generell helse og livskvalitet

Vennligst kryss av den ENE boksen som best beskriver helsen din I DAG.

SMERTER / UBEHAG *

Velg kun en av følgende:

- ◯ Jeg har verken smerter eller ubehag.
- ◯ Jeg har litt smerter eller ubehag.
- ◯ Jeg har middels sterke smerter eller ubehag.
- ◯ Jeg har sterke smerter eller ubehag.
- ◯ Jeg har svært sterke smerter eller ubehag.

© EuroQol Research Foundation. EQ-5D™ is a trade mark of the EuroQol Research Foundation. UK (English) v2.0

*

© EuroQol Research Foundation. EQ-5D™ is a trade mark of the EuroQol Research Foundation

Svar kun på dette hvis følgende betingelser er oppfylt:
0

Generell helse og livskvalitet

Vennligst kryss av den ENE boksen som best beskriver helsen din I DAG.

ANGST / DEPRESJON *

Velg kun en av følgende:

- ◯ Jeg er verken engstelig eller deprimert.
- ◯ Jeg er litt engstelig eller deprimert.
- ◯ Jeg er middels engstelig eller deprimert.
- ◯ Jeg er svært engstelig eller deprimert.
- ◯ Jeg er ekstremt engstelig eller deprimert.

© EuroQol Research Foundation. EQ-5D™ is a trade mark of the EuroQol Research Foundation. UK (English) v2.0

*

© EuroQol Research Foundation. EQ-5D™ is a trade mark of the EuroQol Research Foundation

Svar kun på dette hvis følgende betingelser er oppfylt:
0

Generell helse og livskvalitet

- Vi vil gjerne vite hvor god eller dårlig helsen din er I DAG.
- Denne skalaen er nummerert fra 0 til 100.
- 100 betyr den beste helsen du kan tenke deg.
  0 betyr den dårligste helsen du kan tenke deg.
- Angi på skalaen hvordan helsen din er I DAG ved å dra den grå rundingen oppover/nedover.

HELSEN DIN I DAG =

Norway-Norwegian-EQ-5D-5L
*

Hvert svar må være mellom 0 og 100
Disse feltene kan bare inneholde heltallsverdier.
Vennligst skriv her:

© EuroQol Research Foundation. EQ-5D™ is a trade mark of the EuroQol Research Foundation. Norway (Norwegian) v2.0

*

Søvn

Disse spørsmålene handler om søvn generelt og er ikke nødvendigvis relatert til smertene dine. *

Vennligst velg passende besvarelse til hvert alternativ:

	Sjelden	Noen	Flere ganger

	eller aldri	ganger	per uke
**Hvor ofte har det hendt i løpet av de siste 3 månedene at du har hatt vanskelig for å sovne om kvelden?**	○	○	○
**Hvor ofte har det hendt i løpet av de siste 3 månedene at du har våknet gjentatte ganger i løpet av natten?**	○	○	○
**Hvor ofte har det hendt i løpet av de siste 3 månedene at du har våknet for tidlig og ikke fått sove igjen?**	○	○	○
**Hvor ofte har det hendt i løpet av de siste 3 månedene at du har kjent deg søvnig på dagtid?**	○	○	○

Tanker om smertene (del I)

Hvor engstelig er du for at plagene dine forverres med fysisk aktivitet eller bevegelse? *

Vennligst velg passende besvarelse til hvert alternativ:

Ikke engstelig 0	1	2	3	4	5	6	7	8	9	Veldig engstelig 10
○	○	○	○	○	○	○	○	○	○	○

Tanker om smertene (del II)

1. Hvor mye påvirker smertene livet ditt?
*

Vennligst velg passende besvarelse til hvert alternativ:

Ingen påvirkning 0	1	2	3	4	5	6	7	8	9	Svært stor påvirkning 10
○	○	○	○	○	○	○	○	○	○	○

2. Hvor lenge tror du at smertene dine vil vare? *

Vennligst velg passende besvarelse til hvert alternativ:

Svært kort tid 0	1	2	3	4	5	6	7	8	9	For alltid 10
○	○	○	○	○	○	○	○	○	○	○

3. Hvor mye kontroll føler du at du har over smertene dine? *

Vennligst velg passende besvarelse til hvert alternativ:

	Absolutt ingen kontroll 0	1	2	3	4	5	6	7	8	9	Svært stor kontroll 10
Hvor mye kontroll føler du at du har over smertene dine?	○	○	○	○	○	○	○	○	○	○	○

4. Hvor mye mener du behandling kan hjelpe for smertene dine? *

Vennligst velg passende besvarelse til hvert alternativ:

Ikke i det hele tatt 0	1	2	3	4	5	6	7	8	9	Svært hjelpsom 10
○	○	○	○	○	○	○	○	○	○	○

5. Hvor mye opplever du symptomer fra smertene dine? *

Vennligst velg passende besvarelse til hvert alternativ:

Ingen symptomer i det hele tatt 0	1	2	3	4	5	6	7	8	9	Mange alvorlige symptom 10
○	○	○	○	○	○	○	○	○	○	○

6. Hvor bekymret er du angående smertene dine? *

Vennligst velg passende besvarelse til hvert alternativ:

Ikke bekymret i det hele tatt 0	1	2	3	4	5	6	7	8	9	Svært bekymret 10
○	○	○	○	○	○	○	○	○	○	○

7. Hvor godt føler du at du forstår smertene dine? *

Vennligst velg passende besvarelse til hvert alternativ:

Forstår ikke i det hele tatt 0	1	2	3	4	5	6	7	8	9	Forstår svært godt 10
○	○	○	○	○	○	○	○	○	○	○

8. Hvor mye påvirker smertene dine deg følelsesmessig? (f.eks. gjør den deg sint, redd, urolig eller deprimert)? *

Vennligst velg passende besvarelse til hvert alternativ:

Ikke påvirket følelsesmessig i det hele tatt 0	1	2	3	4	5	6	7	8	9	Svært påvirket følelsesm 10
○	○	○	○	○	○	○	○	○	○	○

Smertemestring

Angi hvor sikker du er på at du kan utføre følgende ting på nåværende tidspunkt, **til tross for smertene**. Husk, spørreskjemaet spør ikke hvorvidt du har gjort disse tingene, men hvor sikker du er på at du kan gjøre tingene, **til tross for smertene**. *

Vennligst velg passende besvarelse til hvert alternativ:

	Helt uenig 0	1	2	3	4	5	Helt enig 6
Jeg kan gjøre ting med glede, til tross for smertene.	○	○	○	○	○	○	○
Jeg kan gjøre det meste av husarbeidet (f.eks. rydde, støvsuge og vaske	○	○	○	○	○	○	○

**opp), til tross for smertene.**							
**Jeg kan være sosial med venner og familie like mye som før, til tross for smertene.**	◯	◯	◯	◯	◯	◯	◯
**Jeg kan håndtere smertene mine i de fleste situasjoner.**	◯	◯	◯	◯	◯	◯	◯
**Jeg kan gjøre noen former for arbeid, til tross for smertene (arbeid inkluderer husarbeid og lønnet/ulønnet arbeid).**	◯	◯	◯	◯	◯	◯	◯
**Jeg kan fortsatt gjøre mange aktiviteter som jeg trives med, f.eks hobbyer, til tross for smertene.**	◯	◯	◯	◯	◯	◯	◯
**Jeg takler smertene mine uten medisiner.**	◯	◯	◯	◯	◯	◯	◯
**Jeg kan fortsatt oppnå de fleste av målene i livet mitt, til tross for smertene.**	◯	◯	◯	◯	◯	◯	◯
**Jeg kan leve et normalt liv, til tross for smertene.**	◯	◯	◯	◯	◯	◯	◯
**Jeg kan gradvis bli mer aktiv, til tross for smertene.**	◯	◯	◯	◯	◯	◯	◯

Mental helse (del I)

Disse spørsmålene handler om følelsene og tankene dine den siste måneden. Marker svaralternativet som korresponderer best med **hvor ofte** du følte eller tenkte i samsvar med det som står i utsagnet. *

Vennligst velg passende besvarelse til hvert alternativ:

	Aldri	Nesten aldri	Noen ganger	Ganske ofte	Svært ofte
**Hvor ofte i løpet av den siste måneden har du blitt irritert over noe som skjedde uventet?**	○	○	○	○	○
**Hvor ofte i løpet av den siste måneden har du følt at du ikke har vært i stand til å kontrollere de betydningsfulle tingene i livet ditt?**	○	○	○	○	○
**Hvor ofte i løpet av den siste måneden har du følt deg nervøs og "stresset"?**	○	○	○	○	○
**Hvor ofte i løpet av den siste måneden har du følt deg trygg på dine evner til å mestre dine personlige**	○	○	○	○	○

problemer?

**Hvor ofte i løpet av den siste måneden har du følt at ting gikk slik som du ønsket det?**	○	○	○	○	○
**Hvor ofte i løpet av den siste måneden har du følt at du ikke kunne rekke over alle tingene du skulle gjøre?**	○	○	○	○	○
**Hvor ofte i løpet av den siste måneden har du vært i stand til å kontrollere irritasjoner i livet ditt?**	○	○	○	○	○
**Hvor ofte i løpet av den siste måneden har du følt at du hadde full kontroll på tingene?**	○	○	○	○	○
**Hvor ofte i løpet av den siste måneden har du blitt sint for ting du ikke hadde innflytelse på?**	○	○	○	○	○
**Hvor ofte i løpet av den siste måneden har du følt at vanskeligheter har hopet seg opp slik at du ikke har vært i stand til å håndtere dem?**	○	○	○	○	○

*

Svar kun på dette hvis følgende betingelser er oppfylt:
! is_empty(PSSPre_SQ002) and ! is_empty(PSSPre_SQ004) and ! is_empty(PSSPre_SQ005) and ! is_empty(PSSPre_SQ010)

Mental helse (del II)

Hvor ofte har du vært plaget med noen av følgende problemer i løpet av **de siste to ukene**? *

Vennligst velg passende besvarelse til hvert alternativ:

	Ikke i det hele tatt	Noen dager	Mer enn halvparten av dagene	Nesten hver dag
**Liten interesse eller glede av å gjøre ting.**	○	○	○	○
**Nedstemthet, depresjon eller følelse av håpløshet.**	○	○	○	○

Generell muskel- og skjeletthelse

Dette spørreskjemaet handler om dine **ledd-, rygg-, nakke-, skjelett- og muskelsymptomer** slik som verking, smerter og/eller stivhet.

For hvert spørsmål, kryss av én boks for å markere hvilket utsagn som best beskriver din situasjon **de siste to ukene.**

1. Smerte/stivhet i løpet av **dagen** *

Vennligst velg passende besvarelse til hvert alternativ:

	Ikke i det hele tatt	Litt	Moderat	Ganske intense	Veldig intense
Hvor intense har dine smerter og/eller stivhet i muskler/ledd vært på dagtid de siste to ukene?	○	○	○	○	○

2. Smerter/stivhet om natten *

Vennligst velg passende besvarelse til hvert alternativ:

	Ikke i det hele tatt	Litt	Moderat	Ganske intense	Veldig intense
Hvor intense har dine smerter og/eller stivhet i muskler/ledd vært om natten de siste to ukene?	○	○	○	○	○

3. Gange *

Vennligst velg passende besvarelse til hvert alternativ:

	Ikke i det hele tatt	Litt	Moderat	Veldig	Har ikke kunnet gå
**Hvor mye har symptomene hindret deg i å gå i løpet av de siste to ukene?**	○	○	○	○	○

4. Vaske seg/påkledning *

Vennligst velg passende besvarelse til hvert alternativ:

	Ikke i det hele tatt	Litt	Moderat	Veldig	Klarer ikke å vaske eller kle på meg
**Hvor mye har symptomene hindret deg i å vaske eller kle på deg de siste to ukene?**	○	○	○	○	○

5. Fysisk aktivitetsnivå *

Vennligst velg passende besvarelse til hvert alternativ:

	Ikke i det hele tatt	Litt	Moderat	Veldig	Ikke i stand til å være fysisk aktiv
Hvor vanskelig har det vært for deg å være så fysisk aktiv som du ønsker (f.eks. å gå en tur eller jogge) pga. ledd-/muskelsymptomene de siste to ukene?	○	○	○	○	○

6. Arbeid/daglige aktiviteter *

Vennligst velg passende besvarelse til hvert alternativ:

	Ikke i det hele tatt	Litt	Moderat	Veldig	Ekstremt
Hvor mye har dine ledd-/muskelsymptomer begrenset deg i ditt arbeid eller daglige aktiviteter de siste to ukene (inkludert jobb og husarbeid)?	○	○	○	○	○

7. Sosiale aktiviteter og hobbyer *

Vennligst velg passende besvarelse til hvert alternativ:

	Ikke i det hele tatt	Litt	Moderat	Veldig	Ekstremt
**Hvor mye har dine ledd-/muskelsymptomer hindret deg i å være sosialt aktiv og å holde på med hobbyer de siste to ukene?**	○	○	○	○	○

8. Behov for hjelp *

Vennligst velg passende besvarelse til hvert alternativ:

	Aldri	Sjeldent	Noen ganger	Ofte	Hele tiden
**Hvor ofte har du trengt hjelp fra andre (inkludert familie, venner eller pleiere) på grunn av ledd-/muskelsymptomene de siste to ukene?**	○	○	○	○	○

9. Søvn *

Vennligst velg passende besvarelse til hvert alternativ:

	Aldri	Sjeldent	Noen ganger	Ofte	Hver natt
**Hvor ofte har du hatt problemer med søvn på grunn av ledd-/muskelsymptomene de siste to ukene?**	○	○	○	○	○

10. Utmattelse eller lite energi *

Vennligst velg passende besvarelse til hvert alternativ:

	Ingen	Litt	Moderat	Veldig	Ekstremt
**Hvor mye utmattelse eller mangel på energi har du følt de siste to ukene?**	○	○	○	○	○

11. Følelsesmessig velvære *

Vennligst velg passende besvarelse til hvert alternativ:

	Ingen	Litt	Moderat	Veldig	Ekstremt
**I hvilken grad har du vært engstelig eller nedtrykt på grunn av dine ledd-/muskelsymptomer de siste to ukene?**	○	○	○	○	○

12. Forståelse av dine helseplager og pågående behandling *

Vennligst velg passende besvarelse til hvert alternativ:

	Fullstendig	Veldig godt	Moderat	Litt	Ikke i det hele tatt
**Når du tenker på ledd-/muskelsymptomene dine; hvor godt føler du at du forstår dine helseplager og din pågående behandling (inkludert din diagnose og medisinering)?**	○	○	○	○	○

13. Tro på å mestre symptomene dine *

Vennligst velg passende besvarelse til hvert alternativ:

	Ekstremt	Veldig	Moderat	Litt	Ikke i det hele tatt
**Hvor sikker har du følt deg i din egen mestring av ledd-/muskelplagene de siste to ukene (f.eks. medisinering eller endring av livsstil)?**	○	○	○	○	○

14. Generell påvirkning *

Vennligst velg passende besvarelse til hvert alternativ:

	Ikke i det hele tatt	Litt	Moderat	Veldig mye	Ekstremt
**Samlet sett, hvor mye har dine ledd-/muskelsymptomer plaget deg de siste to ukene?**	○	○	○	○	○

Sum = {self}

Hvordan er smerteplagene dine nå sammenlignet med før deltagelsen i prosjektet?
*

Velg ett av alternativene
Velg kun en av følgende:

- Veldig mye bedre
- Mye bedre
- Minimalt bedre
- Uforandret
- Minimalt verre
- Mye verre
- Veldig mye verre

Tusen takk for at du svarte på spørreskjemaet!

Hvis du har spørsmål kan du ta kontakt på e-post smartwork@idi.ntnu.no eller ringe [redacted] (Lene Aasdahl, prosjektleder).

Vennlig hilsen
SmaRTWork teamet

Send undersøkelsen.
Takk for at du besvarte undersøkelsen.
