# Supplementary material for: Effectiveness of an artificial intelligence-based self-management intervention to support sick-listed individuals with musculoskeletal disorders in Norway: rationale and protocol for the SmaRTWork randomised controlled trial
Source: BMJ Open. 2026 Aug 18;16(8):e122680. doi: 10.1136/bmjopen-2026-122680 (PMC13504858; doi:10.1136/bmjopen-2026-122680)
Supplement: Supplementary file 3 [file bmjopen-2026-122680.reviewer_comments.pdf]

PEER REVIEW HISTORY

BMJ Open publishes all reviews undertaken for accepted manuscripts. Reviewers are asked to complete a checklist review form and are provided with free text boxes to elaborate on their assessment. These free text comments are reproduced below.

ARTICLE DETAILS

Title (Provisional)

Effectiveness of an artificial intelligence-based self-management intervention to support sick-listed individuals with musculoskeletal disorders in Norway - rationale and protocol for the SmaRTWork randomised controlled trial

Authors

Aasdahl, Lene; Bach, Kerstin; Nilsen, Tom Ivar Lund; Gallina Ottersen, Stuart; Klevanger, Nina E; Eder, Christoph; Øverås, Cecilie Krage; Granviken, Fredrik; Debik, Julia; Lervik, Lars Christian Naterstad; Fossen, Heidi; Bones, Anita; Gismervik, Sigmund; Hara, Karen W; Mork, Paul Jarle

---

VERSION 1 - REVIEW

**Reviewer**	**1**
**Name**	**Gholamrezaei, Ali**
**Affiliation**	**The University of Sydney**
**Date**	**04-Jun-2026**
**COI**	**None**

Authors have provided a detailed trial protocol, explaining the background, rationale, objectives, and methods. The methods are appropriate in addressing the objectives. I have only minor comments:

Methods

Recruitment: please clarify whether exclusion criteria are self-assessed by the participants and/or by the GPs

Development of SmaRTWork: Please mention the number and characteristics of the patients interviewed for the Development of SmaRTWork, diversity is critical to ensure maximum impact

Adherence: Authors mentioned "As participants become more confident in their self-management abilities, they

may reduce or discontinue app use. Therefore, discontinuation is not necessarily a sign of low

compliance but may instead reflect improved self-management." Is tehre any plan to distinguish these during data analysis and reporting?

Outcomes: please clarify who evaluates the primary outcome and how, who makes a final decision whether the primary outcome has been achieved for each individual patient, and whether they will be blinded to the group allocation

Health economic evaluation: please mention if Health economic evaluation will be conducted by someone experienced (or with qualifications) in this field

Strengths and limitations of this study: please add to the limitations that the trial is single-blinded. I understand the feasibility issues but researchers could potentially use a general app to achieve blinding/masking for participants

---

**Reviewer**	**2**
**Name**	**Nim, Casper**
**Affiliation**	**University Hospital of Southern Denmark**
**Date**	**09-Jun-2026**
**COI**	**None**

Thank you for inviting me to review this interesting paper describing a very ambitious project. Overall, this is a well-written and thoughtfully developed protocol. I particularly appreciated the decision to integrate the intervention with SelfBack rather than developing yet another standalone app, as this may improve the likelihood of future implementation and scalability.

I do wonder whether the intervention is likely to have a measurable impact on sick leave, as sick leave is a highly multidimensional outcome that is strongly influenced by social, occupational, and policy-related factors. For example, if Norwegian policies related to sick leave administration through Nav change during the study period, the results may become more difficult to interpret. Nevertheless, meaningful improvements in work participation among people with musculoskeletal disorders are clearly needed, and such challenges should not discourage important research in this area.

Overall, I would like to congratulate the authors on a very interesting project. This is clearly a substantial undertaking, likely supported by a major grant from the Norwegian Research Council, and I look forward to seeing the results.

I have one major comment and a few minor comments that I believe would improve the clarity of the protocol.

Major comment

P. 17, L. 37: “All analyses will be performed by the intention-to-treat principle, and all analyses will be adjusted for factors that are considered predictors of the outcomes under study (e.g., education level).”

The rationale for this adjustment is unclear. Randomization should already address confounding, so it is not obvious what additional purpose the adjustment serves. If the intention is to improve precision rather than reduce bias, this should be stated explicitly. Furthermore, any planned covariate adjustment should be prespecified, and the variables to be included should be clearly defined in the protocol to avoid analytical flexibility. If these details will instead be specified in a separate statistical analysis plan, please clarify this.

Minor comments

• P. 4, L. 34: “adherence to self-management over time without support is challenging for many patients (12)”. I would suggest either toning down this statement, as the cited reference is based on a cross-sectional study, providing a stronger supporting reference, or rephrasing the statement to make it more theoretical.

• P. 5, L. 46: Consider adding “prospectively registered”.

• P. 5, L. 57: A brief description of what is meant by “widespread pain” would be helpful.

• Some of the exclusion criteria appear to be the reciprocal of the inclusion criteria and may therefore be unnecessary.

• The text in Figure 2 is quite small. Consider enlarging the figure and text to improve readability.

• Norway has a rich registry infrastructure for sick leave through Nav. Please clarify whether all outcomes are self-reported or whether any data will be obtained through registry linkage. I assume sick leave status will be measured weekly, but this would benefit from clarification. Additionally, please clarify what is meant by “tailoring” in Table 1.

• How are “pain location” and comorbidity measured?

• Please define NTNU at first mention.

VERSION 1 - AUTHOR RESPONSE

Reviewer: 1
Dr. Ali Gholamrezaei, The University of Sydney
Authors have provided a detailed trial protocol, explaining the background, rationale, objectives, and methods. The methods are appropriate in addressing the objectives. I have only minor comments:
Methods
1. Recruitment: please clarify whether exclusion criteria are self-assessed by the participants and/or by the GPs

Response to comment: We have revised the wording to clarify that eligibility is assessed in two steps: first, a brief self-assessment completed by the participant, followed by the final eligibility assessment conducted by a member of the research team. We have also expanded the recruitment strategy to include additional primary care clinicians who treat patients with musculoskeletal disorders, i.e., physiotherapists, manual therapists and chiropractors. This information is included in the revised manuscript.

2. Development of SmaRTWork: Please mention the number and characteristics of the patients interviewed for the Development of SmaRTWork, diversity is critical to ensure maximum impact

Response to comment: We have added this information to the revised manuscript.

3. Adherence: Authors mentioned "As participants become more confident in their self-management abilities, they may reduce or discontinue app use. Therefore, discontinuation is not necessarily a sign of low compliance but may instead reflect improved self-management." Is there any plan to distinguish these during data analysis and reporting?

Response to comment: We do not plan to perform analyses based on app usage (i.e., per protocol analyses), but we will report on app usage. We have specified this in the revised manuscript.

4. Outcomes: please clarify who evaluates the primary outcome and how, who makes a final decision whether the primary outcome has been achieved for each individual patient, and whether they will be blinded to the group allocation

Response to comment: The primary outcome is derived from registry data. As the dataset will be very large with multiple registrations per participant, the primary outcome will not be assessed individually. Instead, a predefined syntax/algorithm will be used to determine outcome status without individual judgement for each participant. We have clarified this in the revised manuscript. Analyses will be conducted blinded, as described in the Blinding section.

5. Health economic evaluation: please mention if Health economic evaluation will be conducted by someone experienced (or with qualifications) in this field

Response to comment: The health economic evaluation will be led by a health economist. We have specified this is the revised manuscript.

6. Strengths and limitations of this study: please add to the limitations that the trial is single-blinded. I understand the feasibility issues but researchers could potentially use a general app to achieve blinding/masking for participants

Response to comment: We agree and have added this as a limitation.

Reviewer: 2
Dr. Casper Glissmann Nim, University Hospital of Southern Denmark

Comments to the Author:
Thank you for inviting me to review this interesting paper describing a very ambitious project. Overall, this is a well-written and thoughtfully developed protocol. I particularly appreciated the decision to integrate the intervention with SelfBack rather than developing yet another standalone app, as this may improve the likelihood of future implementation and scalability. I do wonder whether the intervention is likely to have a measurable impact on sick leave, as sick leave is a highly multidimensional outcome that is strongly influenced by social, occupational, and policy-related factors. For example, if Norwegian policies related to sick leave administration through Nav change during the study period, the results may become more difficult to interpret. Nevertheless, meaningful improvements in work participation among people with musculoskeletal disorders are clearly needed, and such challenges should not discourage important research in this area.

Overall, I would like to congratulate the authors on a very interesting project. This is clearly a substantial undertaking, likely supported by a major grant from the Norwegian Research Council, and I look forward to seeing the results.
I have one major comment and a few minor comments that I believe would improve the clarity of the protocol.

Major comment
P. 17, L. 37: "All analyses will be performed by the intention-to-treat principle, and all analyses will be adjusted for factors that are considered predictors of the outcomes under study (e.g., education level)." The rationale for this adjustment is unclear. Randomization should already address confounding, so it is not obvious what additional purpose the adjustment serves. If the intention is to improve precision rather than reduce bias, this should be stated explicitly. Furthermore, any planned covariate adjustment should be prespecified, and the variables to be included should be clearly defined in the protocol to avoid analytical flexibility. If these details will instead be specified in a separate statistical analysis plan, please clarify this.

<u>Response to comment</u>: Thank you for pointing out this. In the revised manuscript we have specified that adjustments will be performed to improve precision and that details will be prespecified in the statistical analysis plan.

Minor comments
P. 4, L. 34: "adherence to self-management over time without support is challenging for many patients (12)". I would suggest either toning down this statement, as the cited reference is based on a cross-sectional study, providing a stronger supporting reference, or rephrasing the statement to make it more theoretical.

<u>Response to comment</u>: We have toned down the statement as suggested.

P. 5, L. 46: Consider adding "prospectively registered".

<u>Response to comment</u>: We have specified that the registration was done prospectively in the revised manuscript.

P. 5, L. 57: A brief description of what is meant by "widespread pain" would be helpful. Some of the exclusion criteria appear to be the reciprocal of the inclusion criteria and may therefore be unnecessary.

Response to comment: We have added more information about what is meant by widespread pain. We agree some of the exclusion criteria can be seen as reciprocal to the inclusion criteria, but we would argue that they provide some nuances not covered by the inclusion criteria and have therefore not removed any.

The text in Figure 2 is quite small. Consider enlarging the figure and text to improve readability.

Response to comment: We have enlarged the text in the figure to improve readability.

Norway has a rich registry infrastructure for sick leave through Nav. Please clarify whether all outcomes are self-reported or whether any data will be obtained through registry linkage. I assume sick leave status will be measured weekly, but this would benefit from clarification.

Response to comment: We have specified in the revised manuscript which data are based on registry data from Nav and which is based on questionnaire data. We have also clarified that sick leave data contain exact dates while more long-term benefits are registered monthly.

Additionally, please clarify what is meant by "tailoring" in Table 1.

Response to comment: We have added an explanation in the footnote of the table

How are "pain location" and comorbidity measured?

Response to comment: We have added a more thorough description of these in the revised manuscript. Additionally, the full questionnaire is added to the online supplementary.

Please define NTNU at first mention.

Response to comment: This has been added to the revised manuscript.

VERSION 2 - REVIEW

**Reviewer**	**2**
**Name**	**Nim, Casper**
**Affiliation**	**University Hospital of Southern Denmark**
**Date**	**21-Jul-2026**

COI

Thank you for incorporating my minor comments, I wish you the best of luck with the very exciting RCT, and look forward to seeing the results at conferences and in future publications.
