# Supplementary material for: Effectiveness of an artificial intelligence-based self-management intervention to support sick-listed individuals with musculoskeletal disorders in Norway: rationale and protocol for the SmaRTWork randomised controlled trial
Source: BMJ Open. 2026 Aug 18;16(8):e122680. doi: 10.1136/bmjopen-2026-122680 (PMC13504858; doi:10.1136/bmjopen-2026-122680)
Supplement: Supplementary file 4 [file bmjopen-16-8-s003.docx]

[versjon 04]

Vil du delta i forskningsprosjektet

SMartwork - et digitalt system for persontilpassede anbefalinger for sykmeldte med muskel- og skjelettplager?

## formålet med prosjektet og hvorfor du blir spurt

Dette er et spørsmål til deg om å delta i et forskningsprosjekt hvor vi ønsker å undersøke om en mobilapplikasjon ("SmaRTWork") kan være nyttig for personer som er sykmeldt på grunn av muskel- og skjelettplager. Målet med SmaRTWork appen er å hjelpe deg med å håndtere plagene dine bedre og gi deg relevant informasjon som kan gjøre det lettere for deg å komme tilbake til jobb. Appen er ment å være et ekstra verktøy i tillegg til den oppfølgingen du allerede får fra helsepersonell, arbeidsgiver og NAV. Du blir spurt om å delta i dette prosjektet fordi du er sykmeldt på grunn av muskel- og skjelettplager. Institutt for samfunnsmedisin og sykepleie ved NTNU er ansvarlig for studien.

## Hva innebærer PROSJEKTET for deg?

Vi vil be deg fylle inn et nettbasert spørreskjema om din helsetilstand og bakgrunn. Det tar ca. 10-15 minutter. Etter at du har fylt inn spørreskjemaet vil du ved tilfeldig trekning bli fordelt til en forsøksgruppe eller en kontrollgruppe.

Hvis du fordeles til forsøksgruppen, vil du få tilgang til SmaRTWork appen. For å bruke appen må du aktivere Apple Health (iOS) eller Google Fit/Health Connect (Android) på telefonen din. Dette innebærer at du må akseptere vilkårene til Apple Health (iOS) eller Google Fit/Health Connect (Android) for datadeling. Dette er obligatorisk for deltakelse i studien. Via appen vil du få forslag til hvordan du kan håndtere muskel- og skjelettplagene dine samt informasjon om det å være sykmeldt og prosessen med å komme tilbake til arbeid. Appen kan du bruke så lenge du vil i opptil 12 måneder. For å tilpasse rådene til deg og din situasjon vil du via appen få ukentlige spørsmål om sykmeldingsstatus og hvordan det går med plagene dine. Det tar ca. 1-2 minutter per uke. Hvis spørreskjemaet avdekker utfordringer der en samtale med NAV veileder kan være aktuelt vil en forsker kontakte deg og informere om dette tilbudet. Du velger selv om du vil takke ja til dette. Du kan også bli invitert til et intervju der vi spør om hvordan du har brukt appen og hva som fungerte bra og mindre bra for deg. Deltakelse i intervjuet er frivillig.

SmaRTWork appen er et tillegg til vanlig behandling og skal ikke erstatte kontakt med helsepersonell. Ved spørsmål om din helse tar du kontakt med helsetjenesten slik du ville gjort om du ikke var en del av prosjektet.

Hvis du fordeles til kontrollgruppen vil du ikke få tilgang til å bruke SmaRTWork appen, men deltakere i både forsøksgruppen og kontrollgruppen vil bli bedt om å svare på et spørreskjema etter 3 måneder, 6 måneder og 12 måneder. Dette tar 10-15 minutter hver gang.

I prosjektet vil vi registrere din e-post adresse og ditt telefonnummer. Dette trenger vi for å kunne gi deg tilgang til spørreskjemaene og appen, og for å kunne kontakte deg underveis.

## Innhenting av opplysninger om deg fra nasjonale registre

For å undersøke effekten av appen på sykefravær og gjøre en helseøkonomisk vurdering av SmaRTWork appen trengs det en kobling via ditt personnummer til nasjonale registre for sykefravær (NAV) og helsetjenesteforbruk (Kommunalt pasient- og brukerregister og Norsk pasientregister). Vi vil bruke opplysninger for perioden fra 1 år før du ble med i prosjektet til 1 år etter du ble med i prosjektet, med mulighet for forlengelse opp til 10 år. Taushetsbelagt informasjon som er aktuell å hente fra NAV er bruk av medisinske ytelser (sykemeldinger, arbeidsavklaringspenger og uføretrygd, inkludert gradering, diagnose og datoer), arbeidsrettede tiltak mottatt i regi av NAV, ansettelsesforhold (ansettelsesstatus, stillingsprosent og lønn) og informasjon om og dato for eventuell flytting til utland eller død, mens for helsetjenesteforbruk er det bruk av helsetjenester i kommune- og spesialisthelsetjenesten (inkludert dato for besøk).

## Mulige fordeler og ulemper

Hvis du fordeles til app gruppen vil du få råd om håndtering av plagene dine og veien tilbake til jobb etter sykefravær. Rådene er spesielt rettet mot fysisk aktivitet, styrke- og bevegelighetsøvelser, stressmestring og informasjon om muskel- og skjelettplager. I tillegg vil du få informasjonen om ulike ytelser fra NAV, hva du kan forvente av oppfølging fra NAV, og faktorer i arbeid og privatliv som kan påvirke arbeidsevne og retur til arbeid. Uansett om du fordeles til forsøksgruppen eller kontrollgruppen vil du være med på å gi et viktig bidrag til utvikling av ny kunnskap som kan være til stor hjelp for personer som er sykmeldt for muskel- og skjelettplager. Det er ingen kjent risiko med deltakelse i studien, men fysisk aktivitet kan i sjeldne tilfeller forverre muskel- og skjelettplager. Vanligvis er dette forbigående og smertene vil forsvinne i løpet av få dager. Deltakelse i studien kommer i tillegg til vanlig oppfølging du har av NAV og lege og vil ikke ha noen innvirkning eller konsekvens for annen oppfølging eller behandling du mottar. Hvis du takker ja til å settes i kontakt med din NAV veileder kan det komme frem at andre ytelser enn de du har i dag er mer riktige for deg. Utover dette vil prosjektet ikke påvirke dine ytelser fra NAV. Hvis du har spørsmål underveis i studien kan du kontakte oss på telefon eller via e-post.

## Frivillig deltakelse og mulighet for å trekke ditt samtykke

Det er frivillig å delta i prosjektet. Dersom du ønsker å delta, krysser du av for ja på samtykkeerklæringen på siste side (elektronisk samtykke). Du kan når som helst og uten å oppgi noen grunn trekke ditt samtykke. Det vil ikke ha noen negative konsekvenser for deg eller din oppfølging hos NAV hvis du ikke vil delta eller senere velger å trekke deg. Dersom du trekker tilbake samtykket, vil det ikke forskes videre på dine opplysninger. Du kan kreve innsyn i opplysningene som er lagret om deg, og disse vil da utleveres innen 30 dager. Du kan også kreve at dine opplysninger i prosjektet slettes. Adgangen til å kreve sletting eller utlevering gjelder ikke dersom materialet eller opplysningene er anonymisert. Adgang til sletting kan også begrenses dersom opplysningene er brukt i utførte analyser eller publikasjoner.

Dersom du senere ønsker å trekke deg eller har spørsmål til prosjektet, kan du kontakte prosjektleder (se kontaktinformasjon på siste side).

## Hva skjer med OPPLYSNINGENE om deg?

Opplysningene som registreres om deg skal kun brukes slik som beskrevet under formålet med prosjektet. Eventuelle utvidelser i bruk og oppbevaringstid kan kun skje etter godkjenning fra etisk komité (REK) og andre relevante myndigheter. Du har rett til innsyn i hvilke opplysninger som er registrert om deg og rett til å få korrigert eventuelle feil i de opplysningene som er registrert. Du har også rett til innsyn i sikkerhetstiltakene ved behandling av opplysningene. Du kan klage på behandlingen av dine opplysninger til Datatilsynet og institusjonen sitt personvernombud.

Alle opplysningene vil bli behandlet uten navn og fødselsnummer eller andre direkte gjenkjennende opplysninger (=kodede opplysninger). En kode knytter deg til dine opplysninger gjennom en navneliste. Det er kun prosjektleder som har tilgang til denne listen.

Etter at forskningsprosjektet er ferdig (31.12.2035), vil opplysningene om deg bil oppbevart i ti år for kontrollhensyn. Etter dette vil alle data anonymiseres og oppbevares i inntil 30 år for forskning.

## Forsikring

Deltakelse i studien innebærer ingen kjent risiko for skade. Utover pasientskadeloven er det ikke tegnet egen forsikring for prosjektet. NTNU har et generelt erstatningsansvar hvis skade skulle oppstå.

## Godkjenninger

Regional komite for medisinsk og helsefaglig forskningsetikk ved komiteene for Klinisk utprøving av legemidler og medisinsk utstyr (REK KULMU), har vurdert studien (referansenummer 563919), og har gitt forhåndsgodkjenning.

NTNU og prosjektleder Lene Aasdahl er ansvarlig for personvernet i prosjektet.

Vi behandler opplysningene basert på ditt samtykke og i henhold til NTNU sitt reglement for databehandling.

## kONTAKTOPPLYSNINGER

Dersom du har spørsmål til prosjektet, opplever uønskede hendelser eller bivirkninger, eller ønsker å trekke deg fra deltakelse, kan du kontakte Lene Aasdahl, e-postadresse: lene.aasdahl@ntnu.no.

Dersom du har spørsmål om personvernet i prosjektet, kan du kontakte personvernombudet ved NTNU: personvernombud@ntnu.no.

Jeg samtykker til å delta i prosjektet og til at mine personopplysninger brukes slik det er beskrevet

| Sted og dato | Deltakers signatur |
| --- | --- |
|  |  |
|  | Deltakers navn med trykte bokstaver |
