# Supplementary material for: Effectiveness of an artificial intelligence-based self-management intervention to support sick-listed individuals with musculoskeletal disorders in Norway: rationale and protocol for the SmaRTWork randomised controlled trial
Source: BMJ Open. 2026 Aug 18;16(8):e122680. doi: 10.1136/bmjopen-2026-122680 (PMC13504858; doi:10.1136/bmjopen-2026-122680)
Supplement: Supplementary file 5 [file bmjopen-16-8-s004.docx]

**SmaRTWork – interview guide (English version)**

**1. Background and motivation**

Could you describe your current situation and how it has developed since you first became absent from work? What has influenced your health and your return‑to‑work process?

Could you describe what motivated you to start using the app? What kinds of support or outcomes did you hope it might offer in your situation?

**2. Content and interface**

How did you experience the app's content and interface when you first began using it? How did your experience develop over time?

**3. Relevance**

How did the app align with your needs and preferences?

How well was the content adapted to you personally? What would have increased its usefulness for you?

How did the app’s content relate to the follow‑up you received from health professionals?

**4. Timing**

How did you perceive the timing of when you started using the app? How might earlier or later access have influenced your experience?

**5. Use and influence**

What, if anything, did you make use of from the app, and how did this unfold?

In what ways did it influence your return‑to‑work process, if at all?

**6. Nav**

If NAV became involved as a result of your questionnaire responses, how did you experience that involvement? How did it affect your situation, if at all?

**7. Perspectives on digital tools**

To what extent do you think this type of technology (digital self‑management tools) could be useful in sick‑leave follow‑up?

For whom do you think this type of app may be suitable, and for whom might it be less appropriate?
